# Depositing centromere repeats induces heritable intragenic heterochromatin establishment and spreading in *Arabidopsis*

**DOI:** 10.1093/nar/gkad306

**Published:** 2023-04-24

**Authors:** Zhang-Wei Liu, Jie Liu, Fengquan Liu, Xuehua Zhong

**Affiliations:** Department of Biology, Washington University in St Louis, St Louis, MO 63130, USA; Wisconsin Institute for Discovery & Laboratory of Genetics, University of Wisconsin-Madison, Madison, WI 53706, USA; Department of Biology, Washington University in St Louis, St Louis, MO 63130, USA; Wisconsin Institute for Discovery & Laboratory of Genetics, University of Wisconsin-Madison, Madison, WI 53706, USA; Institute of Plant Protection, Jiangsu Academy of Agricultural Sciences, Nanjing, Jiangsu 210014, China; Department of Biology, Washington University in St Louis, St Louis, MO 63130, USA; Wisconsin Institute for Discovery & Laboratory of Genetics, University of Wisconsin-Madison, Madison, WI 53706, USA

## Abstract

Stable transmission of non-DNA-sequence-based epigenetic information contributes to heritable phenotypic variants and thus to biological diversity. While studies on spontaneous natural epigenome variants have revealed an association of epialleles with a wide range of biological traits in both plants and animals, the function, transmission mechanism, and stability of an epiallele over generations in a locus-specific manner remain poorly investigated. Here, we invented a DNA sequence deposition strategy to generate a locus-specific epiallele by depositing *CEN180* satellite repeats into a euchromatic target locus in *Arabidopsis*. Using CRISPR/Cas9-mediated knock-in system, we demonstrated that depositing *CEN180* repeats can induce heterochromatin nucleation accompanied by DNA methylation, H3K9me2, and changes in the nucleosome occupancy at the insertion sites. Interestingly, both DNA methylation and H3K9me2 are restricted within the depositing sites and depletion of an H3K9me2 demethylase IBM1 enables the outward heterochromatin propagation into the neighboring regions, leading to inheritable target gene silencing to persist for at least five generations. Together, these results demonstrate the promise of employing a *cis*-engineering system for the creation of stable and site-specific epialleles and provide important insights into functional epigenome studies and locus-specific transgenerational epigenetic inheritance.

## Graphical Abstract

**Graphical Abstract:**
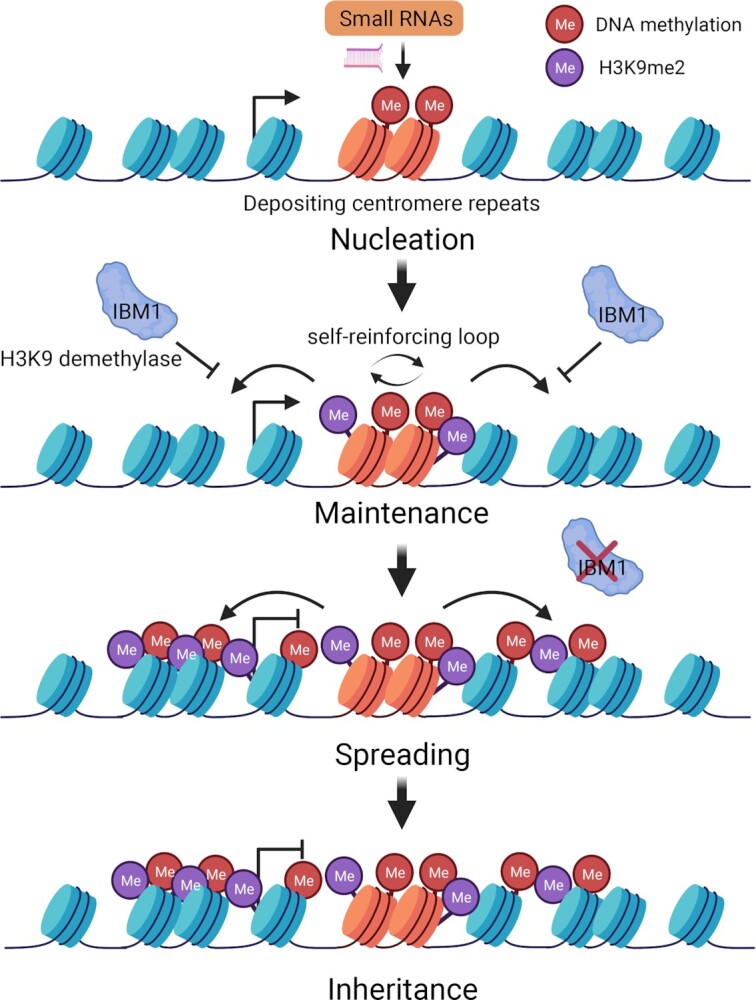
Centromere repeat deposition-induced heterochromatin nucleation, maintenance, and spreading.

## INTRODUCTION

Genetic mutations have long been thought to be the driver of biological complexity and diversity. Emerging evidence indicates that biological complexity continues to evolve even in the absence of genetic variations (1,2). It is largely attributed to epigenetic modifications, which do not affect DNA primary sequences and yet govern the expression of genes and play critical roles in diverse aspects of biological processes, ranging from genome stability to developmental and environmental responses (3–5). The epigenetic variants, referred to as epialleles, are associated with a wide range of biological traits such as behavior adaptation in birds (6), metastable epialleles in mice (7,8), sex determination in fish (9), flowering regulation in plants (10,11), caffeine and oxidative stress induced epimutations in fission yeast (12), and eye color in *Drosophila* (13).

The transgenerational epigenetic inheritance (TEI) of biological traits in epialleles has been well-documented in plants and also some animals (14–16). As with genetic mutations, TEI provides an important path for organisms to develop adaptive responses to environmental stresses and thus has potential implications for breeding and evolution (17). With the exception of few epialleles, most well-known epialleles are spontaneous natural epimutations often, if not always, associated with transposable elements and repetitive DNA sequences (7,17,18). The concept that tandem repeat insertion can induce heterochromatin formation and transcriptional silencing has been well-documented in *Drosophila* and other systems (19–21). The mechanisms by which repetitive DNA elements are targeted for DNA methylation have also been well understood by using the transgene systems in *Arabidopsis* (22,23). However, these studies mainly focus on the insertion sites. It is largely unknown whether the repeat insertion-induced heterochromatic marks can spread and silence the adjacent regions. The spreading mechanism, transmission tempo, and stability over generations also remain to be explored.

The silencing and spreading mechanisms of transposons and other repeats with DNA methylation in promoter and intergenic regions have been well studied (24). Gene promoter methylation represses transcription by directly regulating the binding of transcription factors, and/or indirectly associating with the changes of histone modifications (23). In *Arabidopsis*, approximately 5% of genes contain promoter DNA methylation, whereas the bodies of over one-third of genes are methylated with unclear function (23,25). Gene body methylation (gbM) with mostly CG DNA methylation is often found in constitutively transcribed genes (26). Other type of intragenic methylation with both CG and non-CG methylation in active genes is relatively less studied. While intragenic DNA methylation has been commonly found in diverse plants (27), the function of intragenic DNA methylation in transcriptional regulation and how intragenic heterochromatin is established, maintained, inherited, and erased over time remain largely unknown.

Centromere not only functions to ensure chromosome segregation during cellular division but also plays important roles in genome architecture and chromatin regulation (28). In *Arabidopsis*, the centromere is largely composed of CEN180 repeats (29,30) and highly organized through epigenetic silencing marks such as DNA methylation and histone H3K9me2 (31). Centromeric CEN180 repeats, first found as highly receptive DNA sequences in the *Arabidopsis* genome (32), appear as the main components of the heterochromatin in the centromere (33). Recent studies showed that plant centromere satellite repeats undergo dynamic amplification, expansion, inversions, and adaptation during evolution (34,35). Despite the most abundant DNA repeats in the genome, it remains largely unknown whether depositing centromere satellite repeats into active euchromatic regions can induce chromatin state transition and heterochromatin formation.

Recently developed epigenome editing technology offers promising tools to engineer DNA methylation states in a locus-specific manner (36). Currently, the fusion of epigenetic modifiers with editing machinery such as zinc finger nucleases, transcription activator-like effector nucleases, or deactivated Cas9 has been developed to create an epiallele at a specific site in plants (37–41). However, these methods heavily rely on the enzyme binding efficiency and catalytic activities at the targeted chromatin environment and thus have several limitations such as low efficiency, less specificity, and unstable transmission across generations (38–41). The development of powerful tools to engineer stable epialleles site-specifically by introducing functional DNA elements will be highly valuable.

In this study, we developed a *cis*-regulatory sequence-directed locus-specific epigenome editing strategy with CRISPR/Cas9-mediated deposition of repeat sequences in *Arabidopsis thaliana*. We inserted centromeric 180bp-repeats (*CEN180*) into the 1^st^ intron of euchromatic *ABI5* loci using CRISPR/Cas9-mediated knock-in system and demonstrated that the deposition of two copies of *CEN180* repeats is necessary and sufficient to induce heterochromatin nucleation at the insertion site. DNA methylation and H3K9me2 were positively correlated with the inserting repeat number and were restricted at the insertion sites by an H3K9me2 demethylase IBM1. Knocking out IBM1 can induce the outward heterochromatin spreading leading to *ABI5* silencing that is stable and trans-generationally inherited for at least five generations. The degree of methylation varies dramatically among individual F5 *ABI5* epialleles, causing a wide variation in ABA sensitivity. Collectively, these results demonstrate the promise of employing a *cis*-engineering system for the creation of stable epialleles in a locus-specific manner. Our findings further dissect the mechanism of *CEN180* insertion-induced heterochromatin nucleation and spreading at the intragenic region.

## MATERIALS AND METHODS

### Plant materials

All plants used in the study were of the *Arabidopsis thaliana* Columbia-0 ecotype. The mutant lines used in this study were *abi5* (Salk_200891), *drm1-2* (Salk_031705), *drm2-2* (Salk_150863), *cmt2-7* (WiscDsLox7E02), *cmt3-11* (Salk_148381), *met1-1*, *ddm1-10* (Salk_093009), *nrpd1-3* (Salk_128428), *rdr2-1* (Sail_1277_H08) and *ibm1-5* (Salk_006042C).

Seeds were germinated on $\frac{1}{2}$ MS plates containing 1% sucrose and 0.8% agar after 2 days at 4°C and then transferred to long-day light cycles (16 hours light/8 hours dark) at 22°C. After ∼7–10 days of growth on plates, seedlings were collected for experiments or transferred to soil and grown at 22°C under long-day conditions. For germination assays and scoring ABA sensitivity, 20–100 seeds were plated on $\frac{1}{2}$ MS plates with 1 μM ABA.

### Plasmid construction

The CAS9 vector backbone driven by EC1.2_enhancer_ and EC1.1_promoter_ (42) promoters is from addgene (#71288). The sequences of Pol-III promoters U6-26p, U6-29p, and sgRNA scaffolds were replaced by AAGCTTGGTACCGGGCCCCCATGG sequence. The AtU6 promoter-driven gRNA and donor sequence with the 804bp-left homology arm of *ABI5*, *CEN180*, and 942bp-right homology arm sequences of *ABI5* were constructed in pRI909 (Clotech #3260) as the donor construct. Various copy of *CEN180* repeats were generated by PCR amplification from *Arabidopsis Col-0* genomic DNA. The primers used in plasmid construction are shown in Supplementary Table 2.

### CRISPR/CAS9-mediated knock-in by sequential plant transformation

The fragment knock-in process was performed as previously described (43). The *Arabidopsis Col-0* plants were transformed with plasmids containing an egg-cell specific enhancer and promoter-driven CAS9 enzyme via *Agrobacterium*-mediated floral dipping. After hygromycin selection of T1 plants, immunoblots were used to select a homozygous T2 line with stable expression of CAS9 as the parental line. The donor construct with a sgRNA targeting *ABI5* intron1, *CEN180* repeats, and homology arms were then transformed into this CAS9-containing parental line. Positive T1 lines were selected on 100 mg/l Kanamycin containing MS plates for 7–12 days and transplanted into soil. PCR-based methods were used to identify positive knock-in plants. The siliques of kanamycin resistant T1 plants (one silique per T1 plant) were then pooled together for genotyping. Positive pools were then separated to further genotype for individual positive T1 plants. The homozygous knock-in plants were back-crossed with wild-type *Col-0* to remove the donor and CAS9 transgene.

### Site-specific DNA methylation analysis

Genomic DNA was isolated from one-week plants using CTAB buffer (100 mM Tris–HCl pH8.0, 20 mM EDTA, 1.4 M NaCl, 2% CTAB, 1% PVP). For chop-PCR, 200 ng genomic DNA was digested with McrBC enzyme (New England Biolabs, M0272L) at 37°C for 2 h followed by heat-inactivation of enzyme at 65°C for 20 min. Both digested and undigested DNA was amplified by loci-specific primers.

For bisulfite sequencing of *CEN180* insertion at *ABI5* locus, 500 ng genomic DNA was bisulfite converted by using an EZ DNA Methylation-Gold kit (Zymo Research). The *CEN180* insertion DNA was then amplified by Q5U Hot Start High-Fidelity DNA Polymerase (NEB #M0515). Gel-purified PCR products were cloned into pCR blunt vector (Thermo Fisher Scientific #K270020) and transformed into *E. coli* DH5α competent cells. At least 10 positive clones were sequenced (Genewiz) and analyzed with Kismeth (http://katahdin.mssm.edu/kismeth/revpage.pl).

### Quantitative RT-PCR analysis

Total RNAs were isolated from whole 7-day-old seedlings with or without ABA treatment by using TRIzol reagent (Invitrogen, #15596026). For mRNA expression analysis, after the RNase-free DNaseI (NEB, M0303S) treatment, 300 ng total RNAs were used for reverse transcription by OligodT18VN primer and ProtoScript II (NEB, #M0368L) reverse transcriptase according to the manufacturer's instructions. Quantitative PCR was performed using the CFX96 Real-Time System (Bio-Rad) and SYBR Green Master Mix (Bio-Rad). At least two biological replicates were used for each sample. Gene transcription level was normalized against wild-type *Col-0* and internal control gene *UBQ10*. The primers for RT-qPCR were listed in Supplementary Table 2.

### Small RNA-seq library preparation, sequencing, and analysis

Total RNA extraction was performed by using TRIzol reagent (Invitrogen, #15596026) from 7-day-old seedlings, and dissolved in DEPC-treated H2O. Total RNAs were used for library preparation with Real Seq Bioscience RealSeq^R^-AC Kit (500–00012). The final library products were further purified using 6% polyacrylamide gel (Novex™ TBE Gels, EC6265BOX). The 145–160nt products were excised from the gel for sequencing (single end 50 bp) on a NextSeq 2000 machine (Illumina). The small RNA sequencing data were trimmed using Trimmomatic (v.0.39) and then mapped to the pseudo-genome sequence with insertion of 2 × 180 or 5 × 180 in TAIR10 and called small RNA using ShortStack version 3.8.5 (44) with parameter setting "–mincov 1rpm –pad 75 –mismatches 0 –nohp.

### Immunoblots

Total protein was extracted by lysis buffer (50 mM Tris–HCl, pH 7.5, 150 mM NaCl, 0.5 mM EDTA, 0.1% Triton X-100, 0.2% Nonidet *P*-40). The primary antibodies used were beta-ACTIN (Proteintech, 60008-1-lg) and ABI5 (Abcam, ab98831). Chemiluminescence images were developed using ECL substrate (Bio-Rad) plus image dock (GE healthcare, ImageQuant LAS4000).

### Whole-genome bisulfite sequencing library construction and analysis

Genomic DNA was extracted from 1-week-old seedlings by CTAB buffer (100 mM Tris–HCl pH8.0, 20 mM EDTA, 1.4 M NaCl, 2% CTAB, 1% PVP) and fragmented into ∼200–400 bp average size with a Covaris S220 sonicator. Fragmented DNA was end-repaired, 3’ adenylated, and ligated with TruSeq DNA adapters using Illumina TruSeq DNA PCR-Free Library Prep Kit (Illumina, 20015962). BS conversion was performed using the EZ DNA Methylation-Gold Kit (Zymo Research, D5006). Libraries were amplified 10 cycles using Kapa HiFi HotStart Uracil ReadyMix (Kapa Biosystems, KK 2801) and sequenced on a HiSeq 4000 platform (Illumina) with 50 bp single end reads.

Sequencing reads were trimmed using Trimmomatic (v.0.39) (45) and mapped to the pseudo-genome sequence with insertion of 2 × 180 or 5 × 180 in TAIR10 with BSMAP (v.2.90, parameters: -q 20 -v 5 -w 10 -n 1) (46). The software samtools (v.1.9) (47) was then used to remove duplicate reads and keep uniquely mapped reads. Methratio.py in BSMAP was used to quantify the DNA methylation of cytosines. Only cytosines covered by more than 4 reads were kept for further analysis. Both MethylKit package (48) and Fisher's exact test were used to call DMRs, and the DMRs identified by both methods were used for subsequent analysis. DeepTools (v. 3.3.1) (49) was used to generate data for metaplots. The snapshots of track data were made by the IGV (2.8.2) browser (50). Whole genome bisulfite sequencing data of *Col-0* used for snapshots were downloaded from the NCBI GEO as accession number GSM4955650 (51).

### Chromatin immunoprecipitation (ChIP) assay

The H3K9me2 ChIP assay was performed as previously described (52) with slight modifications. Briefly, 2 g 10-days old seedlings were ground into fine powders in liquid N2 and cross-linked in 15 ml nuclei isolation buffer I (10 mM HEPES, pH 8, 1 M sucrose, 5 mM KCl, 5 mM MgCl_2_, 5 mM EDTA, 0.6% Triton X-100, 0.4 mM PMSF, and protease inhibitors) with 1% formaldehyde for 20 min at room temperature. Cross-linking was quenched by adding 2.04 ml glycine to a final concentration of 125 mM. After being filtered through 2 layers of miracloth (Millipore #475855), samples were pelleted and washed with 1 ml of nuclei isolation buffer II (0.25 M sucrose, 10 mM Tris–HCl, pH 8, 10 mM MgCl_2_, 1% Triton X-100, 1 mM EDTA, 5 mM β-mercaptoethanol, 0.4 mM PMSF, and protease inhibitor). Nuclei were then resuspended with 300ul nuclear lysis buffer (50 mM Tris–HCl 8.0, 10 mM EDTA, 1% SDS, 0.1 mM PMSF, protease inhibitor) and kept on ice for 10 min. The lysates were diluted with dilution buffer (1.1%Triton X-100, 1.2 mM EDTA, 16.7 mM Tris–HCl pH 8, 167 mM NaCl, 0.4 mM PMSF and protease inhibitor) to 1ml, and sheared by sonication. Soluble nucleosomes were isolated after centrifugation at maximum speed for 10 min. Human nucleosomes isolated from HEK293 cells expressing H3.1-FLAG-HA were added as spike-in with a 1:50 ratio. The supernatant was incubated with 4 μg H3K9me2 antibody (Abcam, ab1220), H3 antibody (ab1791), H3.1/H3.2 antibody (Active Motif 61629), or CENH3 antibody (ab72001), and then add 40 μl magnetic protein A/G beads (Life Technologies; 10004D) rotating at 4°C overnight. After sequential washes with low salt buffer (150 mM NaCl, 0.1% SDS, 1% Triton X-100, 2 mM EDTA and 20 mM Tris–HCl, pH 8), high salt buffer (500 mM NaCl, 0.1% SDS, 1% Triton X-100, 2 mM EDTA, and 20 mM Tris–HCl, pH 8), LiCl buffer (0.25 M LiCl, 1% Nonidet *P*-40, 1% sodium deoxycholate, 1 mM EDTA, and 10 mM Tris–HCl, pH 8), and TE buffer (10 mM Tris–HCl, pH 8, and 1 mM EDTA), the DNA-protein complex was eluted with ChIP elution buffer (1% SDS and 0.1 M NaHCO_3_) and reverse cross-linked at 65°C for over 6 h. After proteinase K and RNase treatment, ChIP DNA was purified by standard phenol–chloroform method and used for qPCR. ChIP enrichment and input were normalized with spike-in human ZNF793 gene before calculation, respectively.

### Micrococcal nuclease digestion assay

The nuclei purification and cross-link methods followed the same protocol as the ChIP method. The purified nuclei were resuspended by 0.5 ml MNase buffer and divided into two separate 1.5-ml Eppendorf tubes (250 μl per aliquot). The aliquoted nuclei were cleaved at 37°C for 10 min using 1.5 μl of MNase enzyme (N3755-200UN, Sigma). Addition of 2.5 μl EDTA (0.5M) and 2.5 ul EGTA (0.5M) was used to stop MNase digestion. After reverse cross-linking and proteinase K/RNase treatment, DNA was purified by CTAB buffer.

### Quantification, statistical analysis and reproducibility

Statistical analyses were carried out using Excel and GraphPad Prism 8. Data are presented as mean ± s.d. as indicated. All statistical tests used were two-sided. For the immunoblots and micrographs, at least two independent experiments were repeated with similar results.

## RESULTS

### Depositing *CEN180* repeats induces heterochromatin nucleation at *ABI5* loci

**Figure 1.**
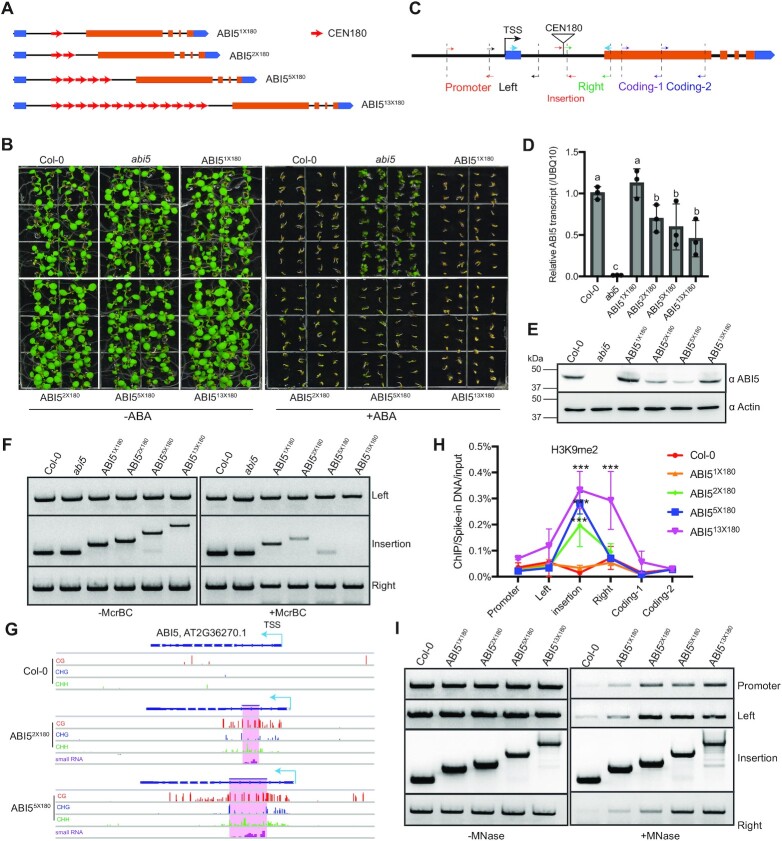
*CEN180* deposition into *ABI5* intron1 induces DNA methylation and H3K9me2. (**A**) Schematic diagrams showing the insertion of various copies of *CEN180* repeats into the first intron of *ABI5* locus. One red arrow represents one *CEN180* repeat. (**B**) Phenotypic analysis showing ABA sensitivity of *Col-0*, *abi5* knockout mutant (Salk_200891), and ABI5^nx180^ knock-in plants with (right) and without (left) ABA treatment. (**C**) Schematic diagram showing the primer location at *ABI5* loci. TSS: transcription start site. Different color of arrows in the related gene region represents different pair of primers used in the following Chop-PCR, ChIP-qPCR, and MNase-based PCR assay at the *ABI5* locus, while the blue arrows represent the primer pair for qRT-PCR. (**D**) Relative *ABI5* transcript level determined by qRT-PCR. Data are mean ± s.d. from three biological replicates. Different letters represent significant differences (*P* < 0.05 by a two-tailed *t*-test) between samples. (**E**) Immunoblot showing the ABI5 protein level using an ABI5 endogenous antibody. Actin serves as a loading control. (**F**) McrBC-based chop-PCR showing the DNA methylation level at the insertion and adjacent regions (left and right) at the *ABI5* locus. (**G**) Snapshots of DNA methylation and small RNA level in *Col-0*, ABI5^2x180,^ and ABI5^5x180^ at *ABI5* loci. The blue arrow represents *ABI5* transcription start site. The shaded area represents the depositing *CEN180* sequences. The DNA methylation data range is [0,1]. (**H**) ChIP-qPCR analysis of H3K9me2 level at various *ABI5* regions. H3K9me2 ChIP samples were first normalized to input, and then to the respective spike-in human chromatin. Data are mean ± s.d. from two biological replicates. Statistical analysis used a two-tailed Student's *t* test (compared with *Col-0*). ***P < 0.001. (**I**) MNase-based PCR assay showing the chromatin accessibility at regions indicated in (C) at *ABI5* locus.

CEN180 satellite repeats, tandemly arrayed with each 180 bp in length (29,30), are the key functional components of *Arabidopsis* centromere (33). We chose *CEN180* repeats as our insertion targets because they are well organized and highly enriched with silent epigenetic marks such as DNA methylation and H3K9me2 (31). We inserted *CEN180* repeats amplified from *Arabidopsis* genomic DNA into the euchromatic *ABA-insensitive 5 (ABI5)* gene using CRISPR-Cas9 knock-in system (detailed in Materials and Methods, Supplementary Figure 1A–E). *ABI5* is chosen because of its important function in the ABA signaling pathway (53,54) and easy visualization and detection of the ABA-insensitive phenotype caused by *abi5* mutation (55). To determine the minimum repeat unit that confers function, we generated *ABI5* knock-in plants carrying 1, 2, 5 and 13 copies (refer as ABI5^1x180^, ABI5^2x180^, ABI5^5x180^, ABI5^13x180^) of *CEN180* repeats (Figure 1A, Supplementary Figure 1C). Sequence alignment showed that *CEN180* repeat sequences in *ABI5* knock-in plants are *CEN180* repeats shared and common to all five *Arabidopsis* chromosomes (Supplementary Data 1, Supplementary Table 1). Sequencing of knock-in plants also confirmed that the insertion points for ABI5^1x180^, ABI5^2x180^, ABI5^5x180^, ABI5^13x180^ are the same as designed CRISPR target (Supplementary Figure 1D). Site-specific PCR and sanger-sequencing were used to verify the *CEN180* copies and sequences in knock-in plants (Supplementary Figure 1C, D). We observed homology-directed repair events at the *ABI5* target with efficiencies ranging from 0.25–1.16% in T1 knock-in plants (Supplementary Table 3).

Further investigation on the T3 homologous lines revealed that all *CEN180* knock-in plants exhibited ABA insensitive phenotype (Supplementary Figure 2A) coupled with decreased *ABI5* transcript and protein level, resembling of the loss of function ABI5 mutant (Supplementary Figure 2B–D). Consistently, we noted that DNAs in all sequence contexts were methylated and small RNAs were accumulated at and surrounding the *CEN180* depositing regions in ABI5^2x180^ and ABI5^5x180^ (Supplementary Figure 2E–J). As a control, we generated a *ABI5* knock-in plant carrying ∼1kb non-repeat scrambled sequence and noted only subtle DNA methylation downstream of the insertion site (Supplementary Figure 2F). Global DNA methylation in ABI5^2x180^ and ABI5^5x180^ plants is similar as the non-repeat control (Supplementary Figure 2K, Supplementary Table 4), suggesting the specificity of DNA methylation establishment at the *ABI5* locus. To exclude the possible effect of donor DNA, we back-crossed the T3 knock-in plants with wild type to remove the CAS9 and donor DNA (Supplementary Figure 1E). Surprisingly, all knock-in plants without donor DNA were sensitive to ABA (Figure 1B) although a slight decrease in *ABI5* transcript and protein level was noted in ABI5^2x180^, ABI5^5x180^, and ABI5^13x180^ plants (Figure 1C–E). These results indicate that the donor DNA induced a transcriptional silencing of *ABI5*. To rule out any possible effect of donor DNA, we backcrossed ABI5^nx180^-Donor plants with *Col-0* and obtained ABI5^nx180^ knock-in plants without donor DNA, which were used for all subsequent studies (Supplementary Figure 1E).

Next, we performed an McrBC-based methylation assay and found increased DNA methylation within the *CEN180* insertion sites in ABI5^1x180^, ABI5^2x180^, ABI5^5x180^, ABI5^13x180^ knock-in plants, and the increased level was in a repeat dosage-dependent manner (Figure 1F). Bisulfite sequencing results further confirmed that DNA methylation particularly CHG and CHH methylation was mostly restricted within the depositing *CEN180* sequences in ABI5^2x180^ and ABI5^5x180^, whereas CG methylation spreads along the intragenic region (Figure 1G). Consistently, the small RNAs were also mainly located within the depositing *CEN180* (Figure 1G, Supplementary Table 4), suggesting that small RNAs were involved in the establishment of intragenic DNA methylation. Similarly, we observed a significant enrichment of repeat-dosage-dependent H3K9me2 at the *CEN180* insertion sites in ABI5^2x180^, ABI5^5x180^ and ABI5^13x180^ (Figure 1H). We further performed the MNase assay to determine the chromatin accessibility and found that depositing more than two copies of *CEN180* repeats can induce chromatin condensation at the insertion adjacent regions (i.e. promoter regions, Figure 1I). This is consistent with the decreased *ABI5* transcript and protein levels in ABI5^2x180^, ABI5^5x180^, and ABI5^13x180^ plants (Figure 1 D, E).

In *Arabidopsis*, histone H3 variant H3.1 is associated with heterochromatin region in the genome, whereas H3.3 is associated with transcriptionally active regions (56) and CENH3 is mainly co-located with CEN180 repeats in centromere region (57). The original *ABI5* gene region is occupied by H3.3 (56). To investigate whether depositing CEN180 at the *ABI5* locus can switch the histone H3 variant loading, we performed H3.1/H3.2 and CENH3 ChIP-qPCR assay and found no enrichment of H3.1/H3.2 and CENH3 at the depositing CEN180 regions at the *ABI5* loci (Supplementary Figure 3A, B). This result suggests that 13 copies of CEN180 repeats are insufficient to recruit CENH3 into the euchromatin, consistent with previous research on mini-chromosomes and ring chromosomes that large CEN180-repeat clusters (approximately 500 kb or longer in length) are needed to possess normal centromere function in *Arabidopsis* (58,59).

Together, these results demonstrated that the deposition of *CEN180* repeats can induce heterochromatin nucleation (i.e. DNA methylation and H3K9me2) at the *ABI5* insertion region and that the efficiency is in a repeat dosage-dependent manner. Small RNA-directed DNA methylation at the *CEN180* insertion sites had a subtle effect on *ABI5* transcription. Deposition of 13 or fewer *CEN180* repeats is insufficient to incorporate CENH3 at the euchromatic loci.

### *CEN180* insertion-induced heterochromatin nucleation requires both CG and non-CG methylation

**Figure 2.**
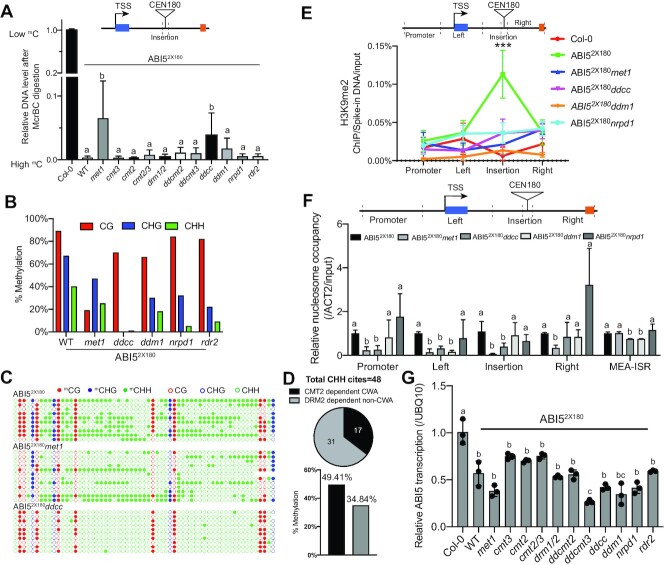
*CEN180* deposition-induced heterochromatin nucleation depends on both CG and non-CG methylation. (**A**) McrBC-qPCR assay showing the relative DNA methylation levels at the *CEN180* insertion region of ABI5^2x180^ knock-in plants in various mutants. All bars represent mean + s.d. from two biological replicates. Different letters represent significant differences (*P* < 0.05 by a two-tailed *t*-test) between samples. TSS: transcription start site. (**B**) The average percentage of DNA methylation level at *CEN180* insertion region determined by bisulfite sequencing. (**C**) Dot plots showing the methylation status of DNA from the individual colony at depositing *CEN180* sequences determined by bisulfite sequencing. Each row represents an independently sequenced clone for each genotype. Solid and open dots represent methylated and unmethylated C, respectively. (**D**) The percentage of CMT2-dependent CWA and DRM1/2-dependent non-CWA methylation at depositing *CEN180* sequences in ABI5^2x180^ plants. The number represents the number of the CHH site at depositing *CEN180* sequences. (**E**) ChIP-qPCR assay showing the H3K9me2 level at various *ABI5* regions. H3K9me2 ChIP samples were first normalized to input, and then to the respective spike-in human chromatin. Data are mean ± s.d. from three biological replicates. Statistical analysis used a two-tailed Student's *t*-test (compared with *Col-0*). ***P < 0.001. (**F**) MNase-qPCR assay showing the nucleosome occupancy at various *ABI5* regions and MEA-ISR (MEDEA-intergenic subtelomeric repeats) sites in the indicated plants. *ACT2* (Actin2) was used as the control. All bars represent mean + s.d. from two biological replicates. Different letters represent significant differences (*P* < 0.05 by a two-tailed t-test) between samples. TSS: transcription start site. (**G**) Relative *ABI5* transcript level in the indicated plants. Data are mean ± s.d. from three biological replicates. Different letters represent significant differences (*P* < 0.05 by a two-tailed *t*-test) between samples.

We next investigated the factors involved in the heterochromatin nucleation of *CEN180* insertion sites by crossing ABI5^2x180^ knock-in plants into various DNA methylation mutants. We found a complete loss of CHG and CHH methylation at the *CEN180* insertion site in *drm1drm2cmt2cmt3* (*ddcc*, a quadruple knockout of all four non-CG methyltransferases) (60) and a strong decrease of CG methylation accompanied with a moderate CHG/CHH reduction in *met1* mutant (CG methyltransferase) (Figure 2A–D, Supplementary Figure 4A). Consistently, we noted that the CHG and CHH methylation were greatly reduced in two small RNA biogenesis-deficient mutants, *nrpd1* and *rdr2* (Figure 2B, Supplementary Figure 4B). Small RNA sequencing data further confirmed that RNA polymerase IV is responsible for the small RNA biogenesis at the depositing CEN180 site (Supplementary Figure 4C). Interestingly, we found that 24nt or >24nt are the main small RNAs that function in the establishment of intragenic DNA methylation (Supplementary Figure 4D, E). Examination of histone marks at the *CEN180* insertion site showed nearly no enrichment of H3K9me2 in *met1* and *nrpd1* mutants (Figure 2E) despite containing an appreciable amount of CHG methylation (Figure 2A–C, Supplementary Figure 4A). This is distinct from the previously established H3K9me2-CHG methylation feedback loop (61), suggesting that CG methylation and small RNAs may also be involved in the establishment of H3K9me2 at the *CEN180* depositing site. We then examined the nucleosome positioning and found that the *CEN180* insertion-induced nucleosome occupancy was significantly reduced in *met1* and *ddcc* mutants at both the insertion and adjacent regions (Figure 2F). Interestingly, the mutation in the chromatin remodeler DDM1, which exhibited a significant decrease of DNA methylation and H3K9me2 at the *CEN180* insertion site (Figure 2A, B and E, Supplementary Figure 4B), only showed nucleosome impairment at the adjacent regions (Figure 2F). This is consistent with a recent report that CG and CHG methylation have a larger impact on chromatin accessibility than small RNA-mediated CHH methylation (62), whereas DDM1 remodels nucleosomes inefficiently at euchromatic loci. Despite the DNA methylation and H3K9me2 loss, we surprisingly found no significant change in *ABI5* transcript levels in *met1, ddcc*, and *nrpd1* mutants (Figure 2G), suggesting that DNA methylation and H3K9me2 over *CEN180* repeats alone are insufficient to regulate *ABI5* transcription in ABI5^2x180^ plants.

Together, these results demonstrated that *CEN180* insertion-induced DNA methylation, H3K9me2, and changes in nucleosome occupancy depend on both CG and non-CG DNA methyltransferases.

### H3K9 demethylase IBM1 blocks heterochromatin spreading at *CEN180* insertion sites

Heterochromatin can spread along chromosomes from the nucleation site in a DNA sequence-independent manner (63). In the ABI5^2x180^ plants, both DNA methylation and H3K9me2 were restricted to the *CEN180* insertion sites and unable to spread to the adjacent regions to fully silence *ABI5* (Figure 1D–G), suggesting that certain factors may block the heterochromatin propagation at the *ABI5* loci. Yeast JmjC domain protein, Epe1, is a putative histone H3K9me demethylase and is required for centromeric heterochromatin integrity by preventing heterochromatin spreading at sites lacking known boundary elements (64,65). In *Arabidopsis*, IBM1 (Increase in BONSAI methylation) is an H3K9me2 demethylase targeting the ectopic H3K9me2 regions in the genome and its loss of function induces gene-body DNA hypermethylation and severe developmental defects (66,67).

**Figure 3.**
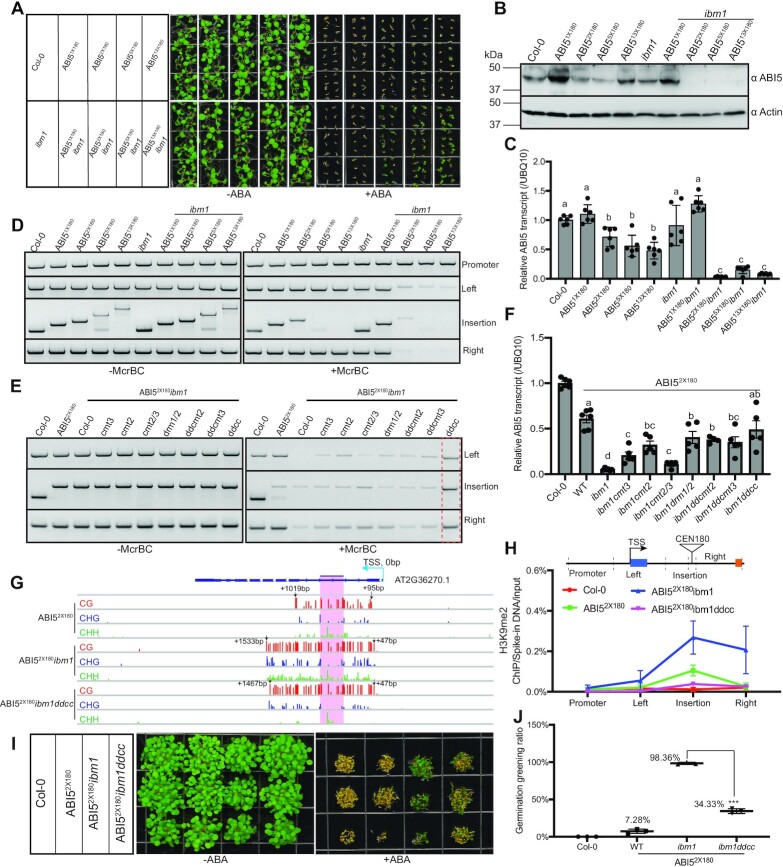
H3K9 demethylase IBM1 blocks intragenic DNA methylation and H3K9me2 propagation. (**A**) Phenotypic analysis showing ABA sensitivity of *ABI5* knock-in plants with or without IBM1. (**B**) Immunoblot showing ABI5 protein level in *ABI5* knock-in plants. Actin serves as an internal control. (**C**) Relative *ABI5* transcript level normalized to *UBQ10* determined by RT-qPCR. Data are mean ± s.d. from six biological replicates. Different letters represent significant differences (*P* < 0.05 by a two-tailed *t*-test) between samples. (**D, E**) McrBC-based chop-PCR showing the DNA methylation level at the various *ABI5* regions. (**F**) Relative *ABI5* transcript level normalized to UBQ10 determined by RT-qPCR. Data are mean ± s.d. from five biological replicates. Different letters represent significant differences (*P* < 0.05 by a two-tailed *t*-test) between samples. (**G**) Snapshot showing the DNA methylation levels of ABI5^2x180^, ABI5^2x180^*ibm1* and ABI5^2x180^*ibm1ddcc* at the *ABI5* locus. The numbers indicate the nucleotide distance from the transcription start site (TSS). The shaded area represents the depositing *CEN180* sequences. The data range is [0,1]. (**H**) ChIP-qPCR assay showing the H3K9me2 levels at the various *ABI5* regions in indicated plants. ChIP samples were first normalized to input, and then to the respective spike-in human chromatin. Data are mean ± s.d. from three biological replicates. Statistical analysis used a two-tailed Student's *t*-test (compared with *Col-0*). ***P < 0.001. (**I**) ABA responsiveness phenotypes of ABI5^2x180^*ibm1ddcc* with (right) and without ABA (left). (**J**) Quantification of germination greening rate in the indicated plants. Data are means ± SD from three biological replicates. Statistical analysis used a two-tailed Student's *t*-test. ***P < 0.001.

To test the function of *IBM1*, we crossed the *ibm1* mutant into *CEN180* knock-in plants. While ABI5^1x180^*ibm1* showed ABA sensitive phenotype similar to wildtype *Col-0*, ABI5^2x180^, ABI5^5x180^ and ABI5^13x180^ plants with *ibm1* mutation exhibited ABA insensitivity (Figure 3A). We also found notably decreased *ABI5* transcript and protein levels in ABI5^2x180^*ibm1*, ABI5^5x180^*ibm1* and ABI5^13x180^*ibm1*, but not in ABI5^1x180^*ibm1* plants (Figure 3B, C). This is consistent with the observation that a single copy of *CEN180* repeat was insufficient to induce a high level of DNA methylation and H3K9me2 at the insertion sites (Figure 1F–H), suggesting that heterochromatin spreading depends on preexisting nucleated chromatin. We next examined DNA methylation and found high DNA methylation levels at the adjacent regions in ABI5^2x180^*ibm1*, ABI5^5x180^*ibm1*, and ABI5^13x180^*ibm1*, but not ABI5^1x180^*ibm1* plants (Figure 3D). To further explore whether small RNAs are involved in the *ibm1*-induced heterochromatin spreading, we performed small RNA sequencing in ABI5^2x180^*ibm1* plants and found that small RNAs propagated into the adjacent regions similar as DNA methylation (Supplementary Figure 5A). Intriguingly, while the left border and CEN180 insertion site are mainly enriched with 24nt or >24nt small RNAs, the right border accumulates 22nt, 23nt, 24nt and >24nt small RNAs (Supplementary Figure 5B–E). This observation indicates that there may be unexplored small RNA biogenesis mechanisms in the intragenic heterochromatin spreading.

To identify the DNA methyltransferase(s) responsible for the methylation spreading, we introduced various DNA methyltransferase mutants into the ABI5^2x180^*ibm1* background and found greatly reduced DNA methylation and H3K9me2 levels coupled with *ABI5* transcriptional restoration in ABI5^2x180^*ibm1ddcc* (Figure 3E–H). As a further confirmation, our ABA phenotypic analysis showed that only ∼34% of ABI5^2x180^*ibm1ddcc* plants exhibited ABA insensitivity, significantly lower than the ∼98% in ABI5^2x180^*ibm1* plants (Figure 3I, J), suggesting that non-CG methylation is involved in the heterochromatin spreading in the *ABI5* intragenic region.

Altogether, these results demonstrated that *IBM1* blocks the spreading of *CEN180* insertion-induced DNA methylation and H3K9me2 at *ABI5* loci.

### Heterochromatin spreading is trans-generationally inherited at *CEN180* deposition sites

**Figure 4.**
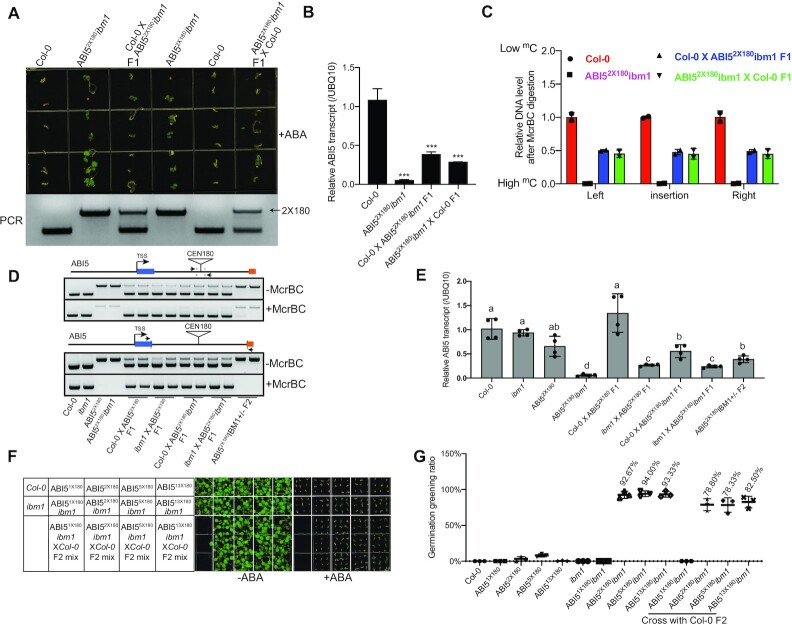
Depletion of IBM1 induces heritable silencing state at *CEN180* depositing *ABI5* loci. (**A**) ABA responsiveness phenotypes of F1 from ABI5^2x180^*ibm1* crossing with *Col-0* (top) and corresponding PCR genotyping (bottom). (**B**) Relative *ABI5* transcript level in the indicated plants. Mean + s.d. from three biological replicates. ***P < 0.001. (**C**) McrBC-based chop-qPCR results showing the DNA methylation level in indicated plants at *ABI5* locus. All bars represent mean ± s.d. from two biological replicates. (**D**) McrBC-based chop-PCR showing the DNA methylation level at the *CEN180* insertion site of the *ABI5* locus. (**E**) Relative *ABI5* transcript level in indicated plants determined by qRT-PCR. Data are mean ± s.d. from four biological replicates. Different letters represent significant differences (*P* < 0.05 by a two-tailed *t*-test) between samples. (**F**) ABA responsiveness phenotypes of ABI5^nx180^*ibm1* and F2 progeny from ABI5^nx180^*ibm1* crossing with *Col-0* with (right) and without ABA treatment (left). (**G**) Quantification of germination greening rate in indicated plants. Data are means ± s.d. from three biological replicates. The number shows the average germination greening rate in indicated plants.

In *Arabidopsis*, transgenerational inheritance has been documented for several TEs and their neighboring genes, such as *BONSAI* (68) and *FWA* (69). Given that depletion of IBM1 can induce the spreading of DNA methylation and H3K9me2 (Figure 3) at the *ABI5*, we examined whether these heterochromatic marks can be stably inherited upon the reintroduction of IBM1. We crossed the ABI5^2x180^*ibm1* with wildtype *Col-0* and found that the majority of F1 progenies were insensitive to ABA treatment accompanied by partially restored *ABI5* transcript and DNA methylation levels compared with ABI5^2x180^*ibm1* (Figure 4A–E), suggesting that *ibm1* induced heterochromatin spreading can be inherited. The inheritance is biparental because F1 progenies from the recipe cross between ABI5^2x180^*ibm1* and *Col-0* showed a similar phenotype (Figure 4A–E). Interestingly, 78.8%, 78.3%, and 82.5% F2 progeny of ABI5^2x180^*ibm1* x *Col-0*, ABI5^5x180^*ibm1* x *Col-0*, and ABI5^13x180^*ibm1* x *Col-0* crosses, respectively, exhibited *ABI5* silencing related ABA insensitive phenotype (Figure 4F, G), suggesting that *CEN180* insertion-induced *ABI5* silencing in *ibm1* mutant is likely a non-mendelian inheritance.

**Figure 5.**
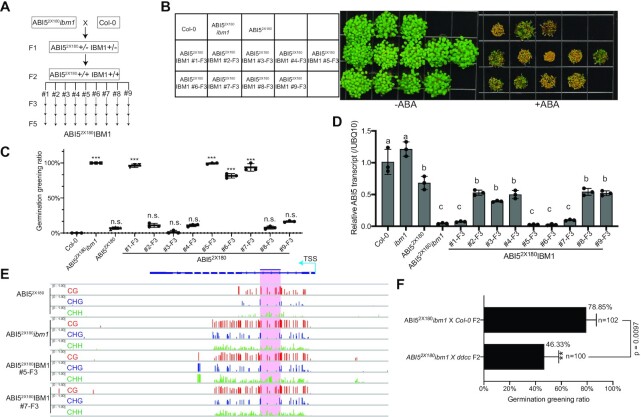
*CEN180*-induced DNA methylation spreading and *ABI5* repression are trans-generationally inheritable. (**A**) Schematic diagram showing the genetic background in the progeny of ABI5^2x180^*ibm1* crossing with *Col-0*. +/+ represents the homozygous plants with ABI5^2x180^ or wild type IBM1; +/– represent the heterozygous plants. (**B**) Phenotypic analysis showing ABA sensitivity of nine (#1–9) individual F3 progenies with ABI5^2x180^IBM1 background from ABI5^2x180^*ibm1* and *Col-0* cross indicated in (A). (**C**) Quantification of germination greening rate of plants shown in (B). Data are means ± s.d. from three biological replicates. Statistical analysis used a two-tailed Student's *t*-test. ***P < 0.001. n.s., no significant. (**D**) Relative transcript level of *ABI5* in the indicated plants. Data are mean ± s.d. from three biological replicates. Different letters represent significant differences (*P* < 0.05 by a two-tailed *t*-test) between samples. (**E**) Snapshot showing DNA methylation levels at *ABI5* locus in indicated plants. The pink shaded area represents the depositing *CEN180* sequences. (**F**) Quantification of germination rate of F2 progeny from ABI5^2x180^*ibm1* crossing with *Col-0* or *ddcc*. Data are means + s.d. from three biological replicates. Statistical analysis used a two-tailed Student's *t*-test. **P < 0.01.

Next, we focused on the F3 progenies containing homozygous ABI5^2x180^ and wild-type IBM1 (ABI5^2x180^ IBM1) (Figure 5A). We found that 4 out of 9 (ABI5^2x180^IBM1 #1-F3, #5-F3, #6-F3 and #7-F3) showed ABA insensitive phenotype and low *ABI5* transcript level (Figure 5B–D) accompanied with moderate DNA methylation levels at the insertion adjacent regions, similar as ABI5^2x180^*ibm1* (Supplementary Figure 6A). This observation suggests that *ibm1* mutation-induced DNA methylation and *ABI5* silencing state can be trans-generationally inherited even after the re-introduction of *IBM1*. As a further confirmation, we determined the genome-wide DNA methylation from the two representative epialleles (ABI5^2x180^IBM1 #5-F3 and #7-F3) and found the maintenance of CG, CHG and CHH methylation at the *ABI5* loci, to a similar extent as ABI5^2x180^*ibm1* (Figure 5E). Besides *ABI5*, we found 266 and 354 CHG hypermethylated regions in ABI5^2x180^IBM1 #5-F3 and #7-F3, respectively, compared to the ABI5^2x180^ parental plants despite the similar global CHG methylation (Supplementary Figure 6B, C). This observation suggests that while the majority of *ibm1*-induced DNA hypermethylation spreading is unstable, DNA methylation at certain loci (i.e. *ABI5*) can be trans-generationally inherited.

Further investigation of F5 generation revealed that while all 8 tested ABI5^2x180^IBM1 #5-F5 progenies maintained the DNA methylation and *ABI5* silencing state, the ABI5^2x180^IBM1 #7-F5 allele demonstrated a phenotype segregation with some progenies had decreased DNA methylation and increased *ABI5* transcription (Supplementary Figure 7A–D). Similarly, H3K9me2 was efficiently maintained in F3 progenies of ABI5^2x180^IBM1 #5 and #7 (Supplementary Figure 5E). Surprisingly, ABI5^2x180^IBM1 #7-F5-2 maintained high H3K9me2 levels at *ABI5* loci despite the loss of DNA methylation (Supplementary Figure 7F), suggesting that the inheritance of DNA methylation and H3K9me2 might be mediated through different mechanisms. Since non-CG methylation is responsible for the heterochromatin spreading (Figure 3E–J), we crossed ABI5^2x180^*ibm1* plants with *ddcc* mutant and found that 46.33% of the F2 plants were insensitive to ABA, significantly less than that of F2 progeny of ABI5^2x180^*ibm1* backcross with *Col-0* (Figure 5F). Together, these results showed that *CEN180* insertion-induced heterochromatin silencing state at *ABI5* can be inherited at least for five generations.

### *CEN180* insertion induced *ABI5* epiallele is trans-generationally inherited

**Figure 6.**
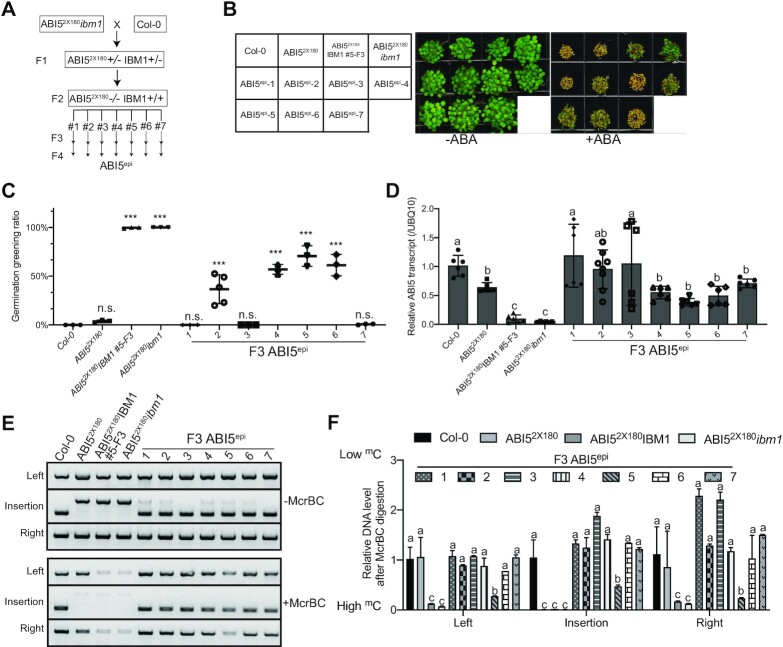
Depositing *CEN180* induces epialleles at the *ABI5* loci. (**A**) Schematic diagram showing the genetic background in the progeny of ABI5^2x180^*ibm1* crossing with *Col-0*. +/+ represents the homozygous plants with ABI5^2x180^ or wild type IBM1; -/- represents the plants without ABI5^2x180^. ABI5^epi^, genetically the same as *Col-0*, shows the *ABI5* epialleles. (**B**) Phenotypic analysis showing ABA sensitivity of seven F3 ABI5^epi^ indicated in (A). (**C**) Quantification of germination greening rate of F3 *ABI5* epialleles showed in (B). Data are means ± s.d. from three biological replicates. Statistical analysis used a two-tailed Student's *t*-test. ***P < 0.001. n.s., no significant. (**D**) Relative *ABI5* transcript level in the indicated plants showed in (B). Data are mean ± s.d. from six biological replicates. Different letters represent significant differences (*P* < 0.05 by a two-tailed *t*-test) between samples. (**E**) McrBC-based chop-PCR showing the DNA methylation level at the various *ABI5* regions in F3 plants indicated in (B). (**F**) McrBC-qPCR assay showing the relative DNA methylation levels at various *ABI5* regions in F3 plants indicated in (B). All bars represent mean + s.d. from three biological replicates. Different letters represent significant differences (*P* < 0.05 by a two-tailed *t*-test) between samples.

To further understand the stability of these epialleles, we removed the *CEN180* repeats by backcrossing ABI5^2x180^*ibm1* into wild-type *Col-0* and investigated F3 progenies without *CEN180* insertion and with wild-type IBM1 (Figure 6A). We named it as ABI5^epi^, which is genetically identical to the *Col-0*. We noted that 3 out of 7 ABI5^epi^ lines (ABI5^epi^-4, ABI5^epi^-5, and ABI5^epi^-6) showed mild ABA insensitive phenotype (Figure 6B, C) coupled with moderate DNA methylation level and *ABI5* transcriptional repression (Figure 6D–F). However, the phenotype is not as strong as the plants with *CEN180* insertion (Figure 5B–D). Further examination of F4 progenies of ABI5^epi^-5 revealed a range of phenotypic variation with ∼1/3 of plants showing the inheritance of the *ABI5* silencing-related ABA insensitive phenotype and DNA methylation (Supplementary Figure 8A–D). This result suggests that the DNA methylation of *ABI5* epiallele although is inheritable but less stable in the absence of *CEN180* repeats.

## DISCUSSION

**Figure 7.**
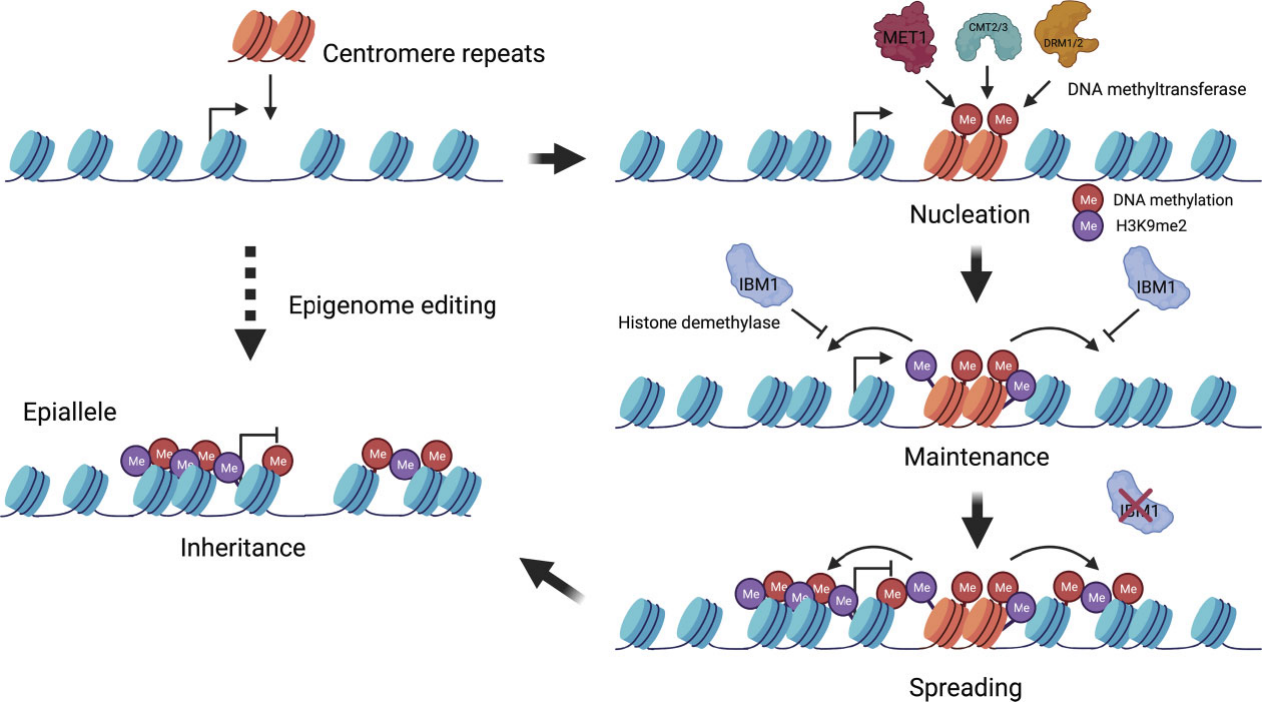
Model of *CEN180* insertion-induced intragenic heterochromatin formation, propagation, spreading, and inheritance. Deposition of *CEN180* satellite repeats into euchromatic gene body regions induces nucleation at the insertion site accompanied with the establishment of DNA methylation and H3K9me2. The heterochromatin state is maintained by the CG methyltransferase MET1 and the self-reinforcing loop between non-CG methylation and H3K9me2. Histone demethylase IBM1 blocks the outward heterochromatin propagation and depletion of IBM1 enables the heterochromatin spreading from depositing sites to adjacent regions, leading to transcriptional silencing. This silencing state is trans-generationally inheritable for at least five generations even in the absence of *CEN180* repeats. This *CEN180* repeat depositing system proves that gene body methylation is functional in heterochromatin formation and transcriptional gene repression and provides new opportunities for epigenetic-based crop improvement. Created with BioRender.com.

Much attention of transgenerational epigenetic inheritance has been given to the natural epigenetic variation, which is mostly attributed to the nearby repeat DNA sequences and transposons. However, the fundamental questions regarding the engineering of locus-specific epigenetic inheritance and the impact of the induced epialleles on organisms remain largely unknown. Here, we developed an epigenome editing approach to engineer a locus-specific epiallele by depositing *CEN180* tandem repeats in a euchromatic locus (Figure 7). Using the CRISPR/Cas9-mediated knock-in system, we demonstrated that DNA repeats with various copies can induce heterochromatin nucleation in the intragenic region and two *CEN180* repeats are necessary and sufficient to induce DNA methylation and H3K9me2 at the insertion sites. The heterochromatin state is maintained by both CG and non-CG methylation but is restricted within the depositing regions by an H3K9me2 demethylase IBM1. Depletion of IBM1 enables outward heterochromatin propagation that is trans-generationally inherited even in the absence of *CEN180* repeats (Figure 7).

One outstanding question regarding the *CEN180* insertion-induced heterochromatin nucleation is how the deposited *CEN180* repeat sequences are first recognized and then recruit the epigenetic machinery to direct *de novo* DNA methylation and H3K9me2 in the *ABI5* locus. Small RNA-deficient plants showed sharply decreased non-CG methylation and H3K9me2 at the *CEN180* insertion site (Figure 2B–D, Supplementary Figure 4B). Small RNA sequencing data further confirmed that these Pol IV-dependent 24nt small RNAs are involved in the establishment of DNA methylation at the depositing *CEN180* site (Figure 1G and Supplementary Figure 4C–E). It suggests that the recognition of *CEN180* sequences at least partially relies on small RNAs, consistent with the well-established concept that small RNAs have a recognized role in defense mechanisms of silencing RNA viruses and transposable elements (70).

Our results revealed that a minimum of two *CEN180* repeat copies is required to initiate the nucleation and the establishment of DNA methylation and H3K9me2 at the insertion site (Figure 1F–I). In *Arabidopsis*, DNA methyltransferases CMT2 and CMT3 are shown to preferentially methylate the dinucleosomal DNA substrates and the maintenance of heterochromatin state involves a self-reinforcing feedback loop between H3K9me2 and DNA methylation (71). This is consistent with our findings that the establishment and maintenance of DNA methylation and H3K9me2 at the *ABI5* locus require at least two *CEN180* copies (∼360 bp) equivalent to two nucleosomes in length.

The incorporation of multiple histone variants with well-known epigenetic mechanisms such as histone and DNA modification plays important roles in the diversity of nucleosome structure and function (72). There are three major histone H3 variants in both plants and animals: DNA replication-coupled canonical H3.1/H3.2, the replacement variant H3.3, and centromere-specific CENH3 (72,73). Only ∼15% of the *CEN180* repeats are bound by CENH3 in the centromere (57), suggesting that large parts of *CEN180* repeats are associated with H3.1/H3.2 or H3.3 in the genome. Our results showed that the deposition of 13× or fewer *CEN180* repeat copies in H3.3-bound euchromatin can induce the establishment of DNA methylation and H3K9me2 (Figure 1F, G) but cannot induce the incorporation of H3.1 and CENH3 (Supplementary Figure 3A, B). It indicates that 13× *CEN180* arrays would be too small and insufficient to exchange the histone H3 variants and form a functional centromere structure in the genome, which is consistent with previous research on *Arabidopsis* mini-chromosomes and ring chromosomes (58,59).

H3.3 variant incorporated in the euchromatic region, associated with actively expressed genes, involves in regulating gene body DNA methylation in *Arabidopsis* (74). Depositing *CEN180* repeats induced heterochromatin formation in the H3.3-bound region but cannot spread adjacently and fully silence the target *ABI5* gene (Figure 1B–E), suggesting that there are unexplored mechanisms that restrict the DNA repeat-directed gene silencing in the gene coding region. In *S. pombe*, H3K9 demethylase Epe1 counteracts RNAi- and H3K9 methyltransferase-mediated heterochromatin maintenance and inheritance (75), revealing a read-and-write mechanism in propagating epigenetic information independent of DNA sequences (76,77). Interestingly, a recent study identified an important role of DNA elements on epigenetic memory of pre-existing H3K9 methylation in the heterochromatin (78), demonstrating a DNA-sequence-dependent epigenetic propagation mechanism. In this study, we showed that depositing *CEN180* repeats can induce nucleation with a high level of DNA methylation and H3K9me2 only at the insertion site, but not the surrounding regions (Figure 1B–G). Deletion of histone H3K9 demethylase IBM1 enables outward heterochromatin propagation and allows the maintenance of DNA methylation and H3K9me2 for several generations (Figure 3A–D). The DNA methylation spreading includes all CG, CHG and CHH sequence contexts (Figure 3G), suggesting that this heterochromatin propagation is DNA sequence independent. Thus, our result supports the reader-writer coupling model of heterochromatin maintenance and spreading through the cell division (63).

In *Arabidopsis*, the establishment of H3K9me2 and non-CG DNA methylation depends on a self-reinforcing reader-writer loop for the stable heterochromatin maintenance (60,79), while CG methylation is established and maintained by *Variant In Methylation* (VIM) proteins and MET1 (80). Of the three methylation contexts, methylation in CG dinucleotides is considered as the most prone to transgenerational inheritance (81). Here, we showed that non-CG methylation can also be trans-generationally inherited. We further revealed an important function of *IBM1* in restricting both DNA and H3K9 methylation within transcribed gene regions (Figure 3E-J). An intriguing observation is that *ibm1*-induced heterochromatin spreading is terminated at the transcription start site (Figure 3G, H), suggesting the existence of other factors or boundary elements in preventing further heterochromatin propagation. It will be interesting to investigate whether there are spreading boundaries mediated by other histone marks, transcription factors, or chromatin remodelers in moderating the heterochromatin propagation along the chromosome.

Paramutation describes as *trans*-homolog interactions that lead to inheritable epigenetic changes in the gene regulation (82). Depositing *CEN180* repeats in the *ABI5* intron region induces *cis CEN180* small RNA-dependent epigenetic silencing marks, which cannot spread adjacently and silence *ABI5* (Figure 1). Depletion of H3K9 demethylase IBM1 induces locus-specific paramutation behaviors (Figures 5 and 6). IBM1 mutation enables the small RNA, DNA methylation, and H3K9me2 outward propagation, which leads to the formation of paramutation at *CEN180* depositing *ABI5* loci (Supplementary Figure 5 and Figures 3–5). It suggests that the programmable locus-specific paramutation is highly associated with the self-reinforcing loop that is dependent on both small RNAs and epigenetic silencing marks. The inheritance of the *ABI5* repression phenotype in the descended plants with depositing *CEN180* is much more stable than without *CEN180* (Figure 5–6), suggesting that DNA repeats and associated epigenetic silencing marks play a crucial role in the transgenerational inheritance.

Natural paramutation and epialleles are well-known to use the read-write DNA and H3K9 methylation system to maintain the epigenetic memory across generations (63). However, the key step of epigenome engineering at a euchromatic locus is the initiation of heterochromatin establishment. Currently, the fusion of epigenetic modifiers with DNA recognition domains (i.e. zinc finger, TAL effector, or deactivated CRISPR) has been developed to manipulate locus-specific epigenome engineering (38–41). However, these methods have the limitations of low efficiency and less specificity with high off-target rates. Our *cis* insertion-directed epigenome editing strategy reported in this study is efficient and specific. We have demonstrated that depositing two *CEN180* repeats is sufficient to induce heterochromatin nucleation specifically at the insertion regions. The identification of IBM1 in preventing the heterochromatin spreading into the neighboring regions further supports the editing specificity.

In summary, this study developed an innovative epigenome editing strategy to engineer a locus-specific, inheritable epiallele by depositing functional DNA elements into the non-coding region of targeted genes. This strategy expands the current genome and epigenome editing scope from gene coding region to whole genomic loci including promoter and introns and provides new opportunities for crop improvement and clinical applications.

## DATA AVAILABILITY

All WGBS and small RNA-seq data produced in this study were deposited into Gene Expression Omnibus under accession number GSE201629.

## Supplementary Material

gkad306_Supplemental_Files
