# Supplementary material for: Depositing centromere repeats induces heritable intragenic heterochromatin establishment and spreading in Arabidopsis
Source: Nucleic Acids Res. 2023 Apr 24;51(12):6039–54. doi: 10.1093/nar/gkad306 (PMC10325890; doi:10.1093/nar/gkad306)
Supplement: gkad306_Supplemental_Files [file gkad306_supplemental_files.zip › Supplementary data.pdf]

SUPPLEMENTARY DATA

Depositing centromere repeats induces heritable intragenic heterochromatin establishment and spreading in *Arabidopsis*

Zhang-Wei Liu[1,2], Jie Liu[1,2], Fengquan Liu[3], Xuehua Zhong[1,2*]

[1]Department of Biology, Washington University in St Louis, St Louis, MO, 63130, USA

[2]Wisconsin Institute for Discovery & Laboratory of Genetics, University of Wisconsin-Madison, Madison, Wisconsin, 53706, USA

[3]Institute of Plant Protection, Jiangsu Academy of Agricultural Sciences, Jiangsu Key Laboratory for Food Quality and Safety-State Key Laboratory Cultivation Base of Ministry of Science and Technology, Nanjing, Jiangsu 210014, China

* To whom correspondence should be addressed. Email: xuehuazhong@wustl.edu

Supplementary Figures

Supplementary Figure 1. CRISPR-CAS9 mediated knock-in system and *ABI5* locus.
Supplementary Figure 2. Characterization of *CEN180* knock-in plants with donor DNA.
Supplementary Figure 3. Depositing *CEN180* repeats is insufficient to recruit H3.1/H3.2 and CENH3 in the euchromatic region.
Supplementary Figure 4. *CEN180* insertion-induced DNA methylation depends on both CG and non-CG methyltransferases.
Supplementary Figure 5. Small RNAs spread into adjacent regions in *ibm1* mutant.
Supplementary Figure 6. DNA methylation analysis in F3 progeny with ABI5^{2X180}IBM1 background.
Supplementary Figure 7. *ABI5* silencing state can be tran-generationally inherited with depositing *CEN180* repeats
Supplementary Figure 8. Inheritance of ABI5 epialleles.

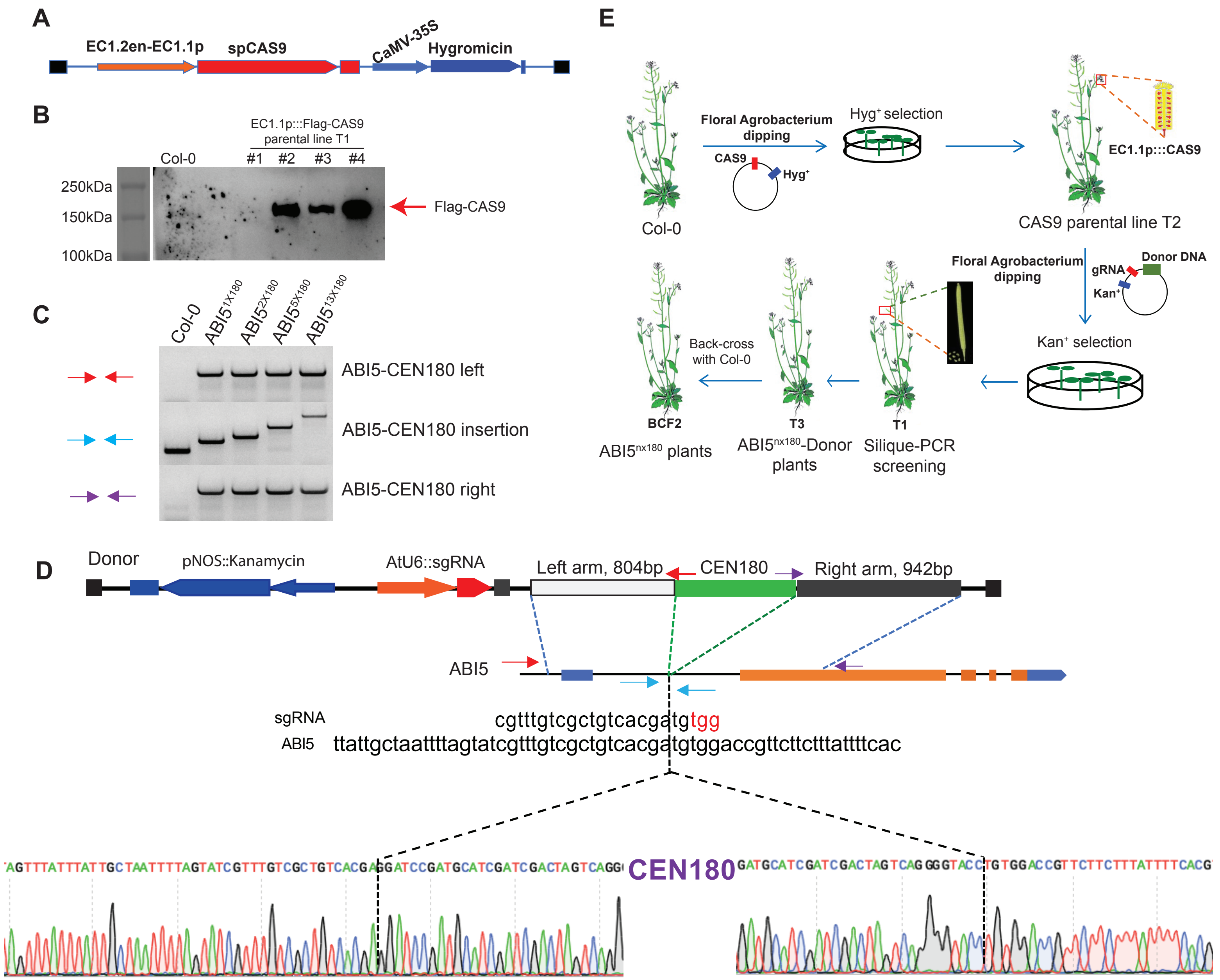

Supplementary Figure 1. CRISPR-CAS9 mediated knock-in system and ABI5 locus

A, Schematic diagram of the vector with cas9 driven by EC1.2 enhancer and EC1.1 promoter. **B,** Western blot showing the Cas9 positive transgenic lines. Cas9 plasmid in (**A**) was transformed into *Col-0*, and the flower buds from individual T1 transgenic plant were used for the western-blot. **C,** Genotyping results showing the positive ABI5-CEN180 knock-in plants. **D,** Schematic showing donor DNA construct, the sgRNA sequences, donor and *ABI5* target site. Red bases indicate the PAM sequences of sgRNA. The arrows represent the primers for knock-in plant detection. **E,** Outline of the sequential transformation strategy and screening procedure for CEN180 knock-in plants. T3 homozygous ABI5^{nx180} plants with donor DNA was back-crossed with *Col-0*, and BCF2 progenies without donor DNA were generated for subsequent studies.

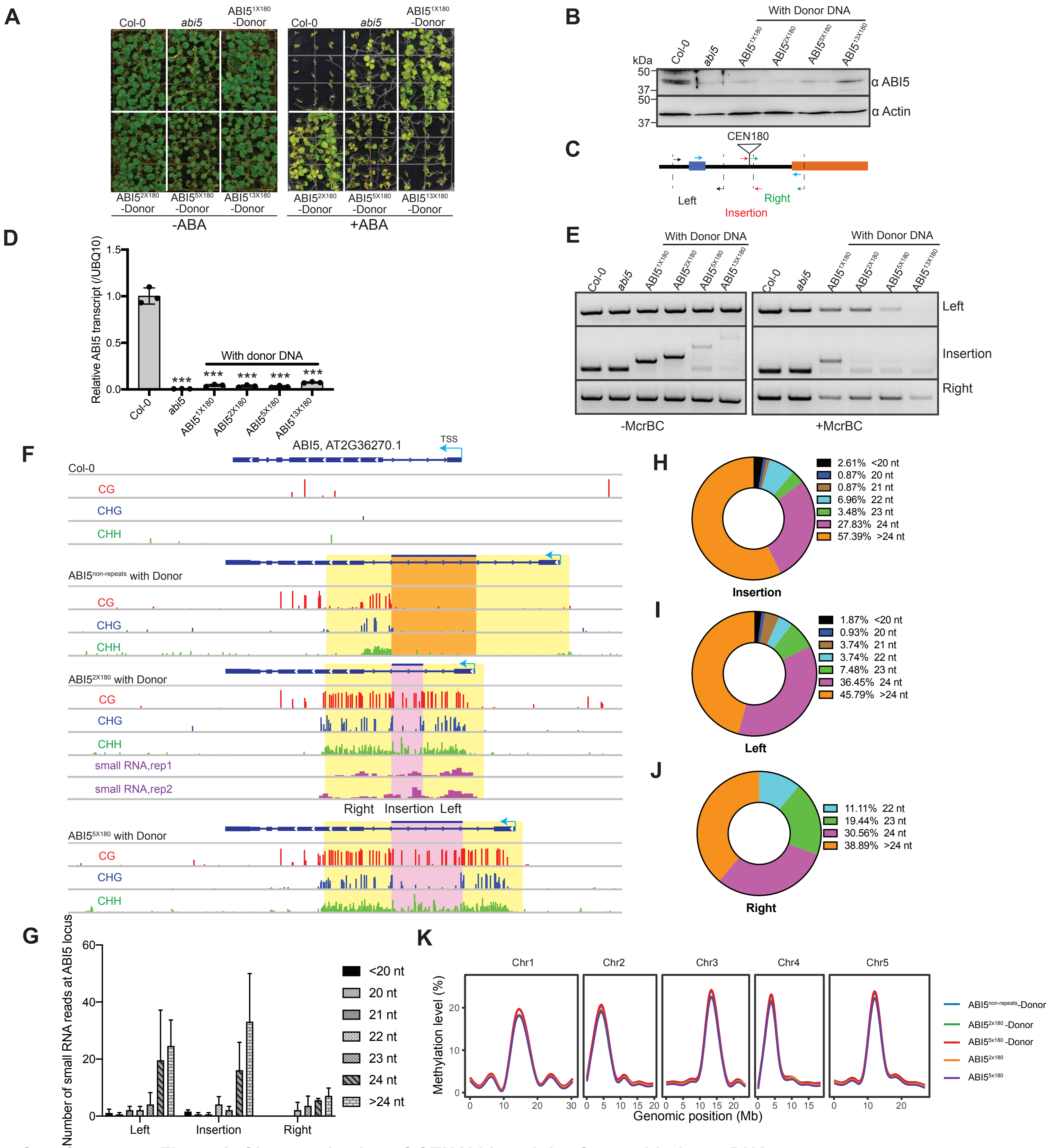

Supplementary Figure 2. Characterization of *CEN180* knock-in plants with donor DNA

A, Phenotypic analysis showing ABA sensitivity of ABI5^{nx180}-Donor with and without ABA treatment. **B,** Immunoblot showing the ABI5 protein level using an ABI5 endogenous antibody. Actin serves as a loading control. **C,** Schematic drawings of the primer location at *ABI5* loci. **D,** Relative *ABI5* transcript level in the indicated plants. Primers showed in **C** with blue arrow. Data are mean ± s.d. from three biological replicates. Statistical analysis used two-tailed Student's t test. ***, $p < 0.001$. **E,** McrBC based chop-PCR showing the DNA methylation level in knock-in lines (with donor DNA) at *ABI5* locus. **F,** Snapshot showing DNA methylation level in *Col-0*, ABI5$^{non-repeats}$-Donor, ABI5^{2X180}-Donor, and ABI5^{5X180}-Donor plants at *ABI5* loci. Yellow shaded area represent the donor DNA sequences, orange shaded area represents the non-repeats sequences, and pink shaded area represents the depositing *CEN180* sequences. The DNA methylation data range is [0,1]. The small RNA level was also showed in ABI5^{2X180}-Donor plants. TSS: transcription start site. **G,** Small RNA sequencing data showing the number of different length of small RNA reads from indicated region in ABI5^{2x180}-Donor shown in **F**. Data are mean + s.d. from two biological replicates. **H-J,** Percentage of different length of small RNAs from indicated region in ABI5^{2x180}-Donor. **K,** Metaplot showing the genome-wide DNA methylation level in indicated plants. Chr1 to Chr5 represent five chromosomes.

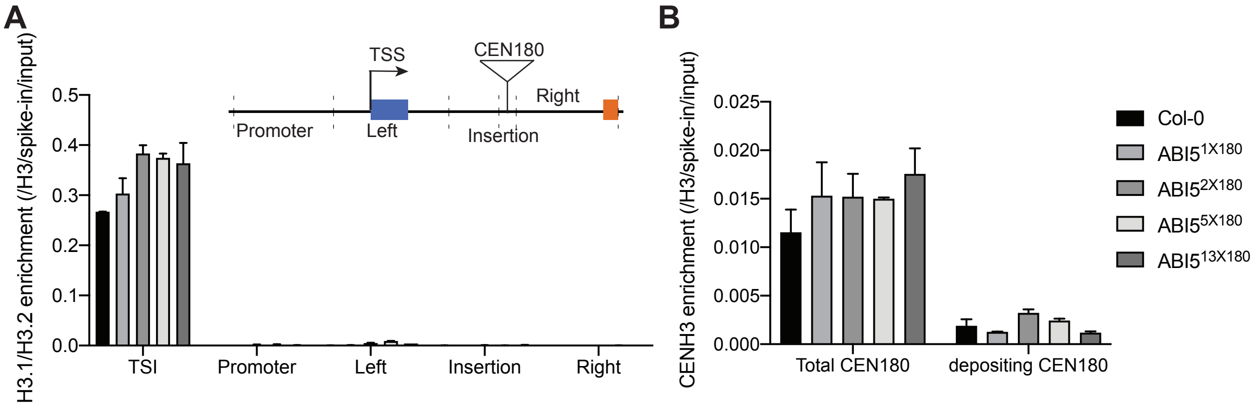

Supplementary Figure 3. Depositing CEN180 repeats is insufficient to recruit H3.1/H3.2 and CENH3 in the euchromatic region.

A, ChIP-qPCR analysis of H3.1/H3.2 enrichment at various *ABI5* regions. H3.1/H3.2 ChIP samples were first normalized to input, and then to total H3 and the respective spike-in human chromatin. Data are mean + s.d. from three biological replicates. TSI (Transcriptionally Silencing Information) loci was used as the positive control for H3.1/H3.2 ChIP. **B,** ChIP-qPCR analysis of CENH3 enrichment at the endogenous centromeric CEN180 loci and the depositing CEN180 at *ABI5* locus. Data are mean + s.d. from three biological replicates.

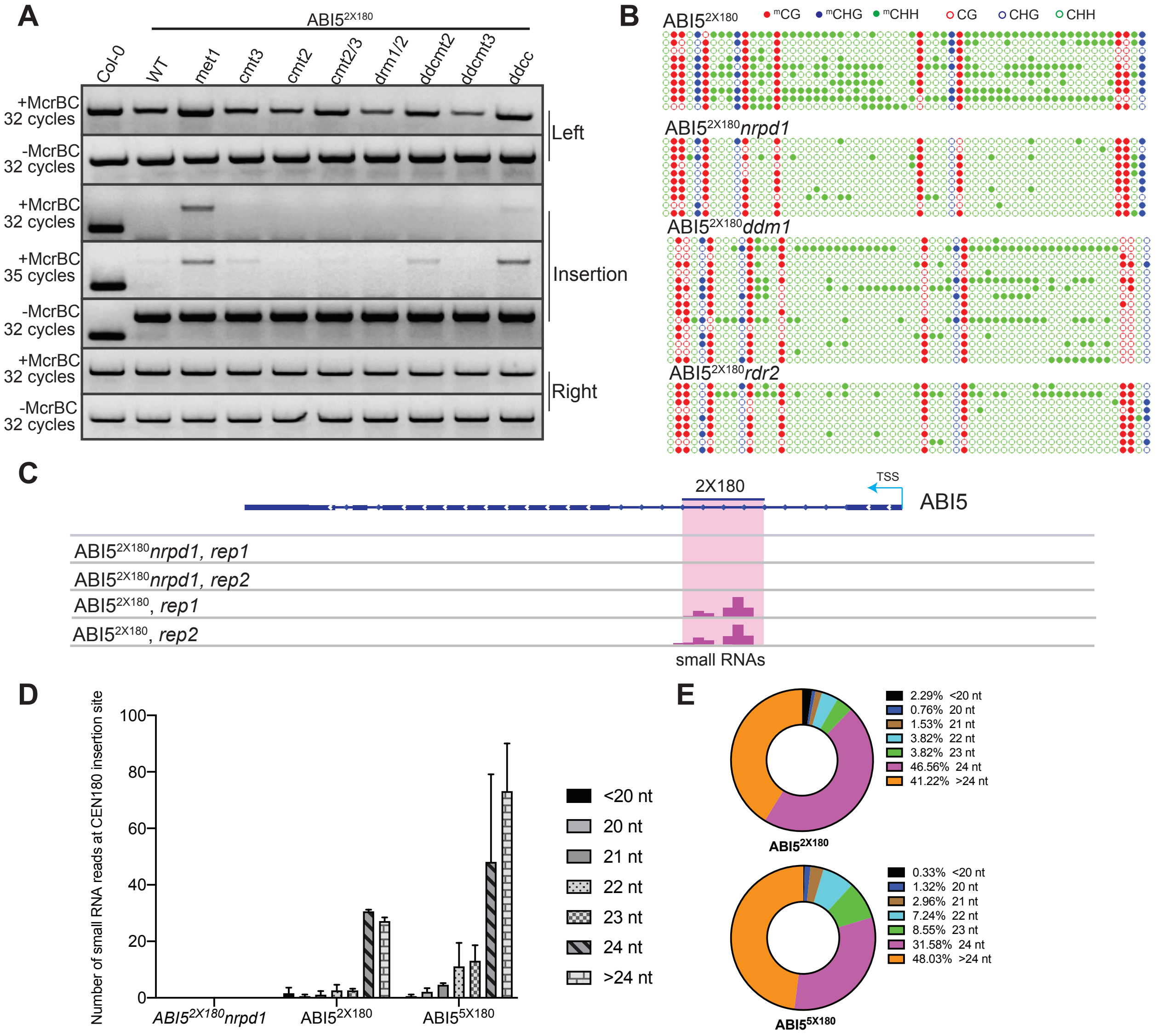

Supplementary Figure 4. *CEN180* insertion-induced DNA methylation depends on both CG and non-CG methyltransferases. A, McrBC based chop-PCR showing the DNA methylation level of the insertion and adjacent regions (left and right) in indicated mutants. **B,** Dot plots showing the methylation status of DNA from the individual colony at depositing *CEN180* site determined by bisulfite sequencing. Each row represents an independently sequenced clone for each genotype. Solid and open dots represent methylated and unmethylated C, respectively. **C,** Snapshots showing the small RNA level in indicated plants. The shaded area represents the depositing *CEN180* sequences. The small RNA data range is [0,40]. TSS: transcription start site. **D,** Small RNA sequencing data showing the number of different length of small RNA reads from indicated depositing CEN180. Data are mean + s.d. from two biological replicates.**E,** Percentage of different length of small RNAs from indicated depositing CEN180.

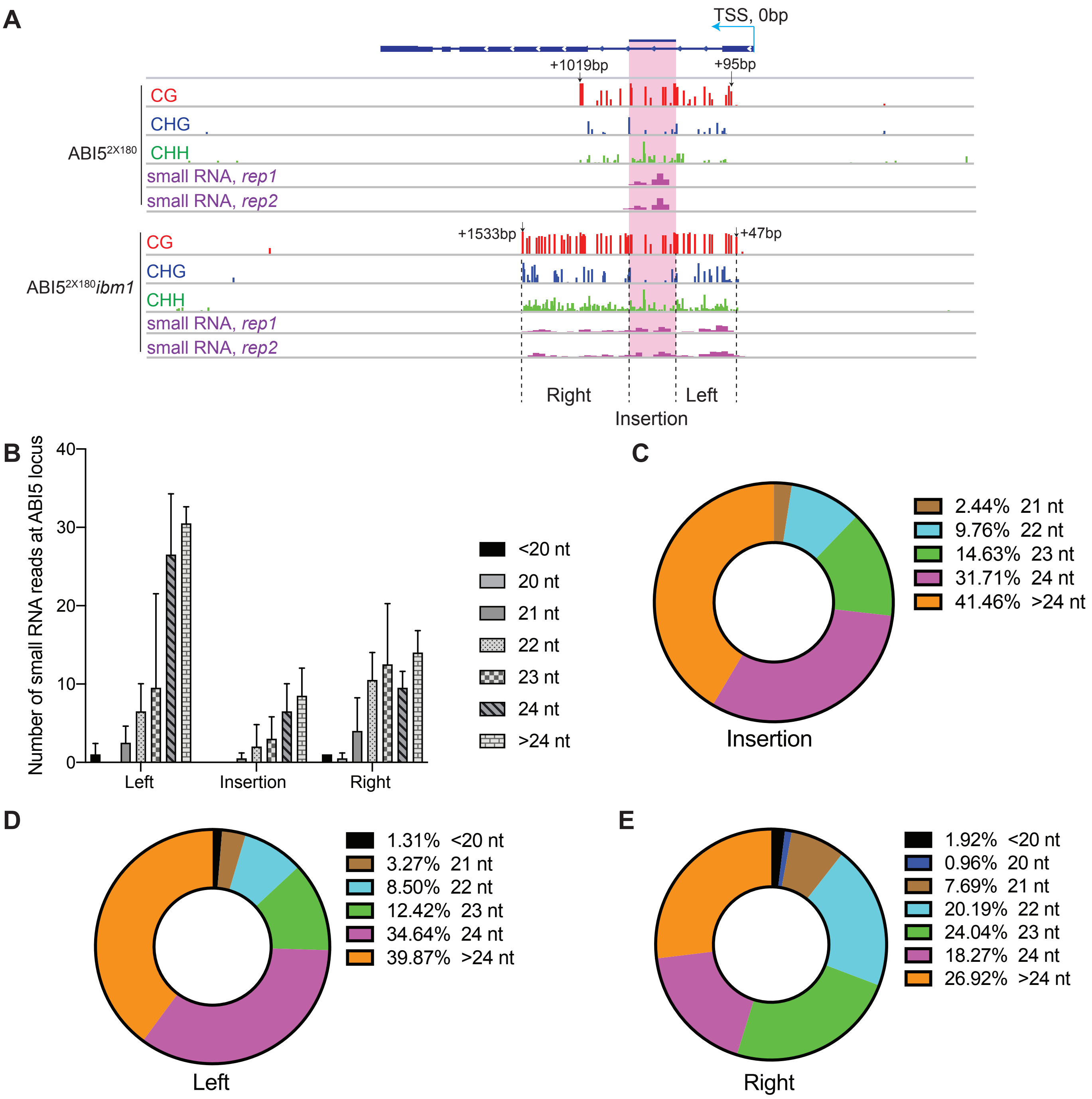

Supplementary Figure 5. Small RNAs spread into adjacent regions in *ibm1* mutant.
A, Snapshots showing the DNA methylation and small RNA levels in indicated plants. The shaded area represents the depositing *CEN180* sequences. The DNA methylation data range is [0,1]. The small RNA data range is [0,40]. TSS: transcription start site. The numbers indicate the nucleotide distance from TSS. **B,** Small RNA sequencing data showing the number of different length of small RNA reads from the indicated region in ABI5^{2x180}*ibm1* plants. Data are mean + s.d. from two biological replicates. **C-E,** Percentage of different length of small RNAs from the indicated region in ABI5^{2x180}*ibm1* plants.

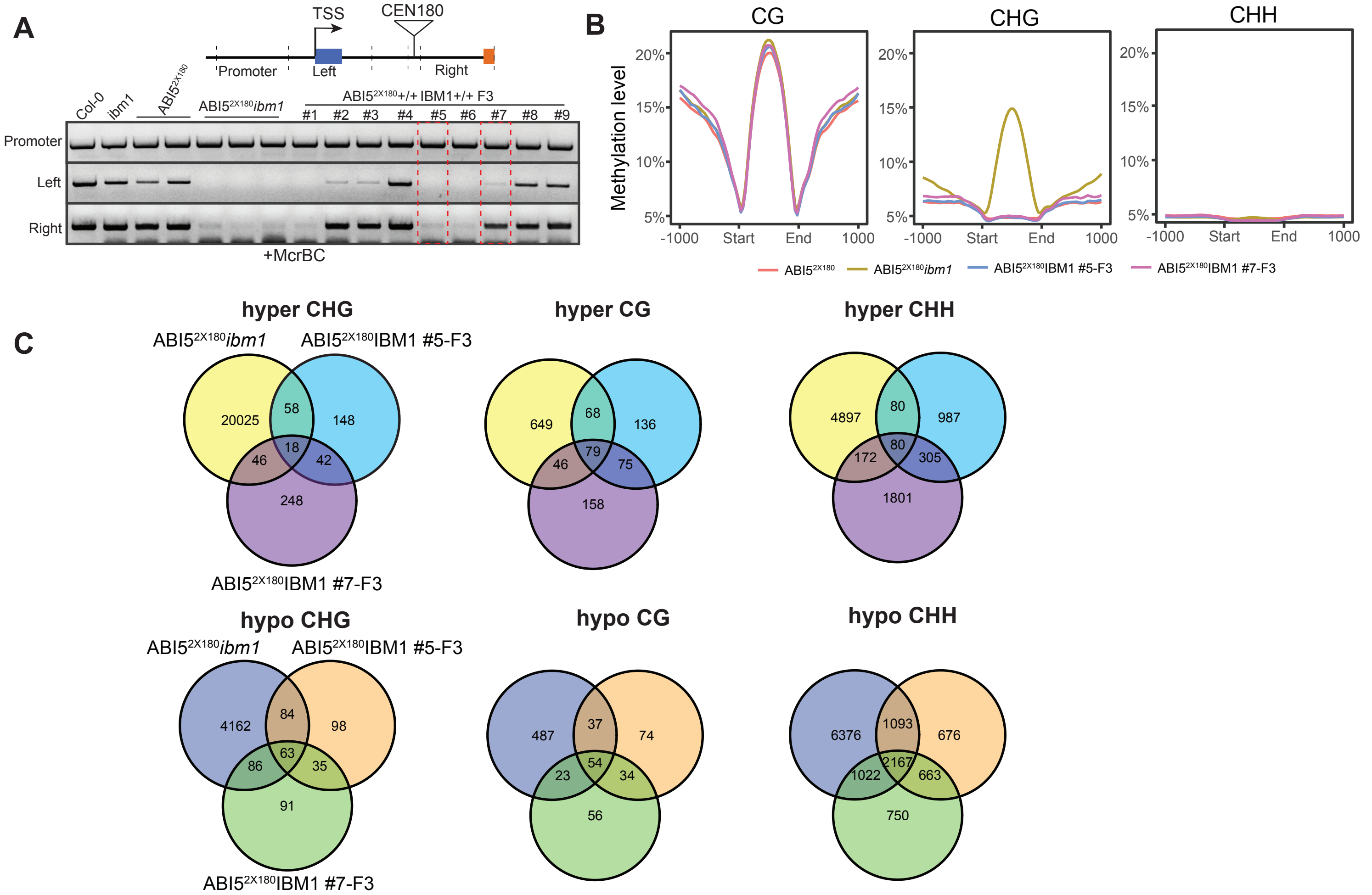

Supplementary Figure 6. DNA methylation analysis in F3 progeny with ABI5^{2X180} IBM1 background.

A, McrBC-PCR assay showing the relative DNA methylation levels at ABI5 locus in the indicated F3 plants from ABI5^{2X180}*ibm1* crossing with *Col-0*. +/+ represents the homozygous plants with ABI5^{2X180} or wild type IBM1. **B,** Metaplot showing the average genome-wide CG, CHG, and CHH methylation level at protein coding genes. "-1000" indicates the upstream 1000 bp of the transcription start site (Start), and "1000" indicates the downstream of 1000 bp of the transcription end site (End). **C,** Venn diagrams showing the overlaps of hyper- and hypo-CG, CHG, or CHH differentially methylated regions (DMRs) between ABI5^{2X180}*ibm1* (yellow, hyper DMR; blue, hypo DMR), ABI5^{2X180}IBM1 #5-F3 (light blue, hyper DMR; orange, hypo DMR), and ABI5^{2X180}IBM1 #7-F3 (purple, hyper DMR; green, hypo DMR).

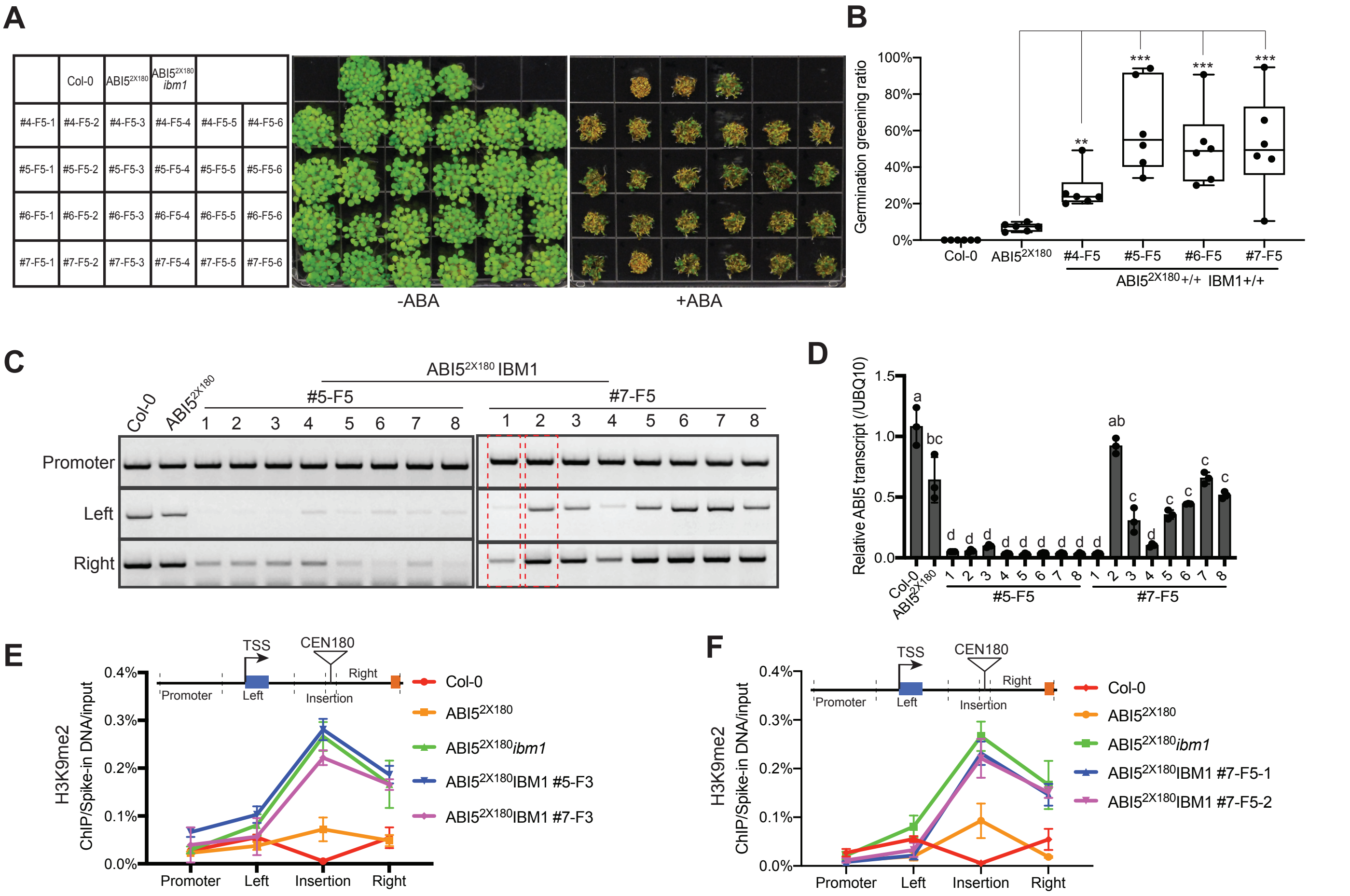

Supplementary Figure 7. ABI5 silencing state can be tran-generationally inherited with depositing CEN180 repeats

A, Phenotypic analysis showing ABA sensitivity of ABI5^{2X180}+/+ IBM1+/+ F5 plants from ABI5^{2X180}*ibm1* crossing with *Col-0*. **B,** Boxblot showing the quantification of germination greening rate of F5 plants in the presence of ABA in **A**. Each dot represents one individule F5 line. Statistical analysis used two-tailed Student's t test. **, p < 0.01.***, p < 0.001. **C,** McrBC-based chop-PCR showing the DNA methylation level at *ABI5* locus in the indicated F5 plants from ABI5^{2X180}*ibm1* crossing with *Col-0*. **D,** Relative *ABI5* transcript level in the indicated plants showed in **C**. Mean ± s.d. from three biological replicates. Different letters represent significant differences (P < 0.05 by a two-tailed t-test) between samples. **E, F,** ChIP-qPCR assay showing H3K9me2 level at different regions of ABI5 locus in the indcated plants.Mean ± s.d. from three biological replicates.

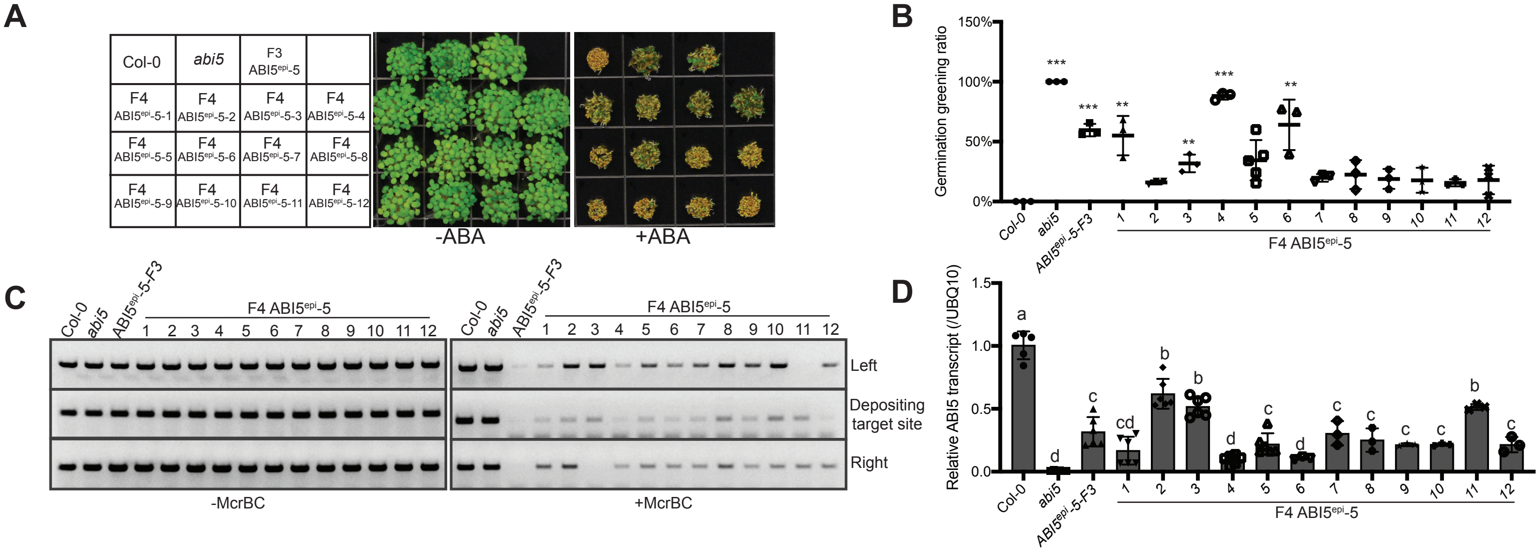

Supplementary Figure 8. Inheritance of *ABI5* epialleles.

A, Phenotypic analysis showing ABA sensitivity of #5-F4 *ABI5* epialleles ($ABI5^{epi}$, genetically same as *Col-0*) from $ABI5^{2X180}$*ibm1* and *Col-0* cross. **B,** Quantification of germination greening rate of #5-F4 ABI5 epialleles showed in **A**. Data are means ± s.d. from three biological replicates. Statistical analysis used two-tailed Student's t test for the difference from Col-0. ***, p < 0.001.**, p < 0.01. **C,** McrBC based chop-PCR showing the DNA methylation level in #5-F4 *ABI5* epialleles (shown in **A**). **D,** Relative transcript level of *ABI5* in the indicated plants. Mean ± s.d.from at least three biological replicates. Different letters represent significant differences (P < 0.05 by a two-tailed t-test) between samples.
