# Supplementary figures and images for: Depositing centromere repeats induces heritable intragenic heterochromatin establishment and spreading in Arabidopsis
Source: Nucleic Acids Res. 2023 Apr 24;51(12):6039–54. doi: 10.1093/nar/gkad306 (PMC10325890; doi:10.1093/nar/gkad306)

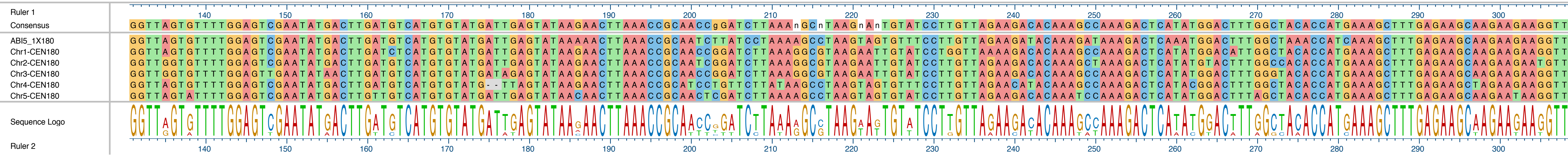

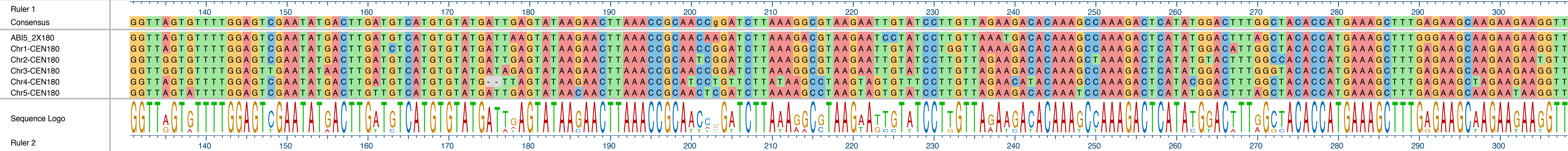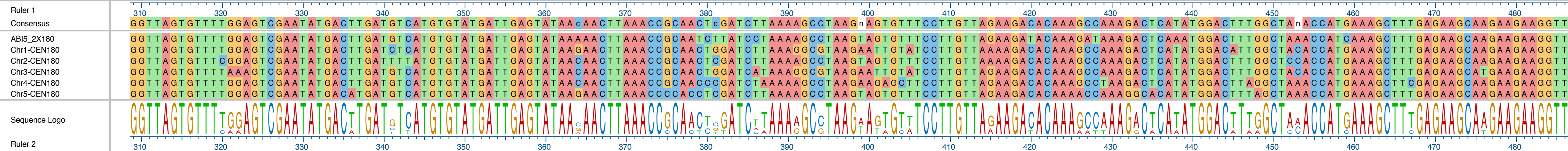

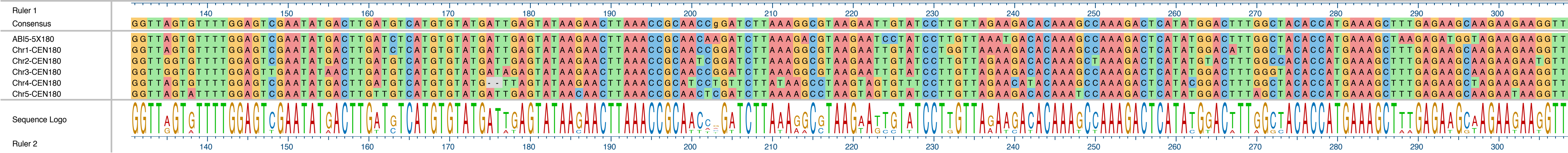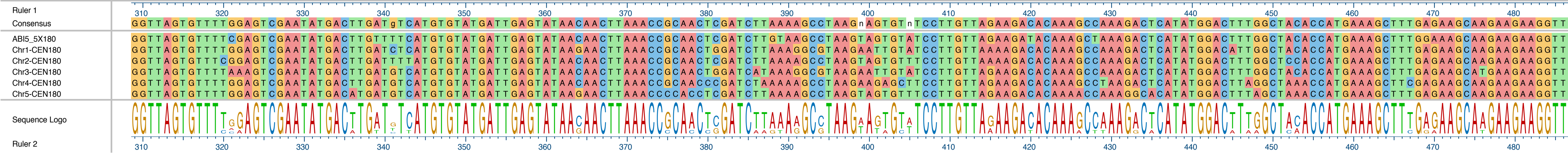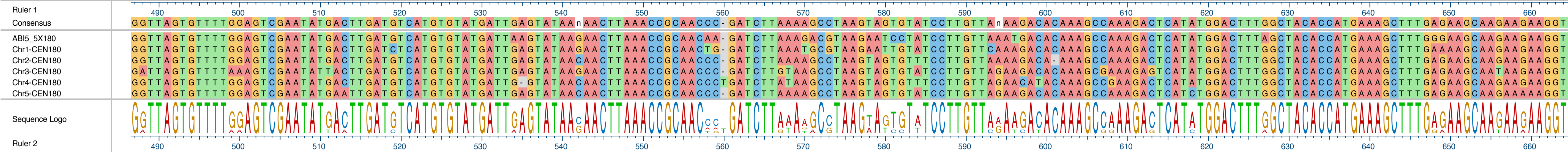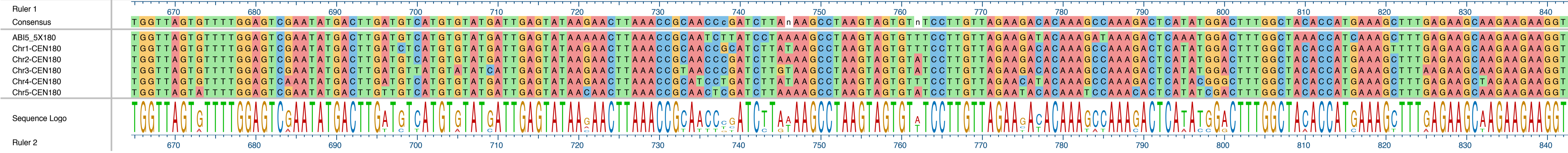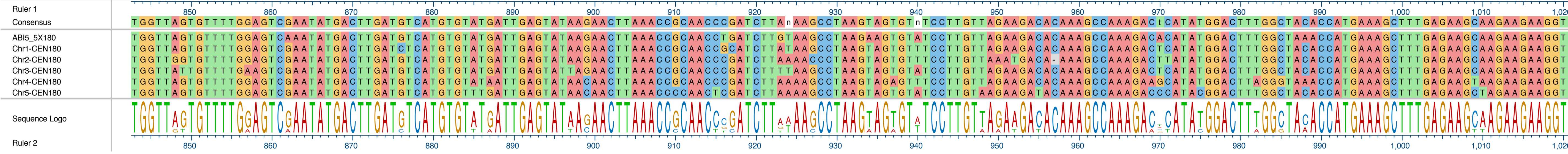

Supplement: gkad306_Supplemental_Files [file gkad306_supplemental_files.zip › Supplementary Data 1 . Depositing CEN180 sequences alignment with original CEN180 in different chromosomes.pdf]
